# Frictional strength regulated by roughness alignment

**DOI:** 10.1126/sciadv.ady6779

**Published:** 2025-09-17

**Authors:** Shaoqi Huang, Shuwen Zhang, Deheng Wei, Hengxu Song, Yifan Li, Jianwei Cheng, Hu Zhao, Siyang Song, Zeqing Li, Liang Li, Shubao Shao, Chongpu Zhai, Minglong Xu

**Affiliations:** ^1^ State Key Laboratory for Strength and Vibration of Mechanical Structure, School of Aerospace Engineering, Xi’an Jiaotong University, Xi’an 710049, China.; ^2^ State Key Laboratory of Intelligent Deep Metal Mining and Equipment, School of Resources and Civil Engineering, Northeastern University, Shenyang 110819, China.; ^3^ State Key Laboratory of Nonlinear Mechanics, Institute of Mechanics, Chinese Academy of Sciences, Beijing 100190, China.; ^4^ School of Engineering Science, University of Chinese Academy of Sciences, Beijing 100049, China.

## Abstract

Rough contacts are ubiquitous, yet the impacts of the roughness matching on frictional behavior remain insufficiently understood. We investigate how slow shear and stick-slip transition depend on the roughness alignment between contacting surfaces. By rotating one surface with respect to the other, we precisely control roughness matching, enabling nearly an order-of-magnitude variation in the maximum static friction coefficient. Both the variation amplitude and the angular range over which the frictional strength varies are governed by roughness alignment. Universal statistical patterns emerge in micromechanical quantities, including the microcontact area, orientation, deformation, and their evolutions under shear, following generalized extreme value distributions. To quantify asperity interactions, we introduce the contact fabric tensor, whose anisotropy provides a unified measure of roughness matching and a predictor for frictional strength and sliding onset. These findings advance our understanding of friction at rough interfaces and inform the design of mechanical systems, adhesives, and fault models.

## Teaser

Matching the textures of contacting rough surfaces alters how they resist and release motion.

## INTRODUCTION

Contacts between rough surfaces are ubiquitous, as real surfaces inherently exhibit multiscale random irregularities, ranging from the nano-sized textures (*1*–*5*) to submicron patterns appearing at machined surfaces (*6*–*10*) to kilometer-scale features on earthquake faults (*11*). Proper alignment of the contact pairs, such as fracture-sealing interfaces (*12*), seismic faults (*13*), biological joints, and soft layers (*14*, *15*), leads to unique frictional properties. Rough-rough contacts are frequently modeled as rough-to-flat contacts for simplification (*8*, *16*, *17*), where the overall interfacial behavior arises from the collective contributions of individual contacting asperities. These discrete microcontacts govern fundamental interfacial and frictional properties, evolving from elastic to plastic deformation under increasing normal loads (*18*). Additional mechanisms have been further integrated, including viscosity (*19*), adhesion (*20*), and breakage (*21*), to interpret the macroscopic behavior of friction (*22*), lubrication (*23*), interfacial stiffness (*24*), etc. Different from tip-flat microcontacts considered in the ideal contact model, real rough-rough interfaces involve a complex interplay of asperities, such as shoulder-to-shoulder, peak-to-valley, and peak-to-shoulder asperity microcontacts (*25*). These oblique microcontacts introduce complex micromechanical effects (*26*), including rolling, slipping, squeezing, bending, and even ploughing under transverse loads. Despite their importance, the role of asperity micromechanics in rough-rough contacts with varying roughness matching levels remains underexplored, leading to inconsistencies in frictional modeling and interpretation.

Normal contact measurements and analyses indicate good agreement between rough-flat and rough-rough scenarios in terms of resultant normal contact forces and pressure distribution, while disparity arises when the shear load is applied (*27*, *28*). As the friction develops between two rough solid surfaces, the interface transitions from static to unstable states, accompanied by local stick-to-slip events occurring at discrete microcontacts formed at contacting asperities (*29*). The static friction force increases until it reaches the frictional strength (*30*), followed by complete interfacial sliding with the coefficient of the dynamic friction typically lower than that of the static friction (*31*). The development of friction from stick-slip to fully sliding regimes is affected by both material properties and evolving interface geometry (*32*). The difference between static and dynamic friction is usually attributed to the growth of the real contact area between rough surfaces, arising from contact aging (*8*) and slipping weakening (*33*, *34*). During the stick-slip transition, the microcontact development is observed to be anisotropic and direction dependent, with both the leading and trailing edges of a microcontact slipping under increasing shear stress (*35*–*37*). The onset of the sliding is dictated by the preslip stress distribution and the amount of potential energy accumulated, which can experience abrupt change at rupture fronts (*38*–*40*). When it comes to rough-rough contacts, the interactions among asperities of varying shapes, sizes, and orientations become more complex, as they compete with or complement material properties in determining frictional behavior. These asperity interactions evolve spatially and temporally during the stick-slip transition, directly contributing to the accumulation, release, and dissipation processes of the interfacial energy. Rough contact pairs with different roughness matching levels tend to show distinct frictional strength, transition, and stability behavior (*34*, *41*). To quantitatively assess the role of roughness alignment, rigorous three-dimensional (3D) analyses that capture detailed asperity-level micromechanics are desired.

This study examines how roughness matching influences frictional behavior by comparing contact pairs with controlled isotropic roughness and varied roughness matching levels. Through slow friction experiments combined with in situ 3D x-ray computed tomography (3DXRCT) tests under incrementally increasing tangential loads, we capture the stick-slip transition, contact structure evolution, and interfacial micromechanics. To quantify roughness matching and its impact on friction, we introduce the contact fabric tensor, which integrates asperity-scale interactions and provides a predictive framework for frictional strength at rough interfaces.

## RESULTS

### Dependence of stick-slip friction on roughness matching

**Fig. 1.**
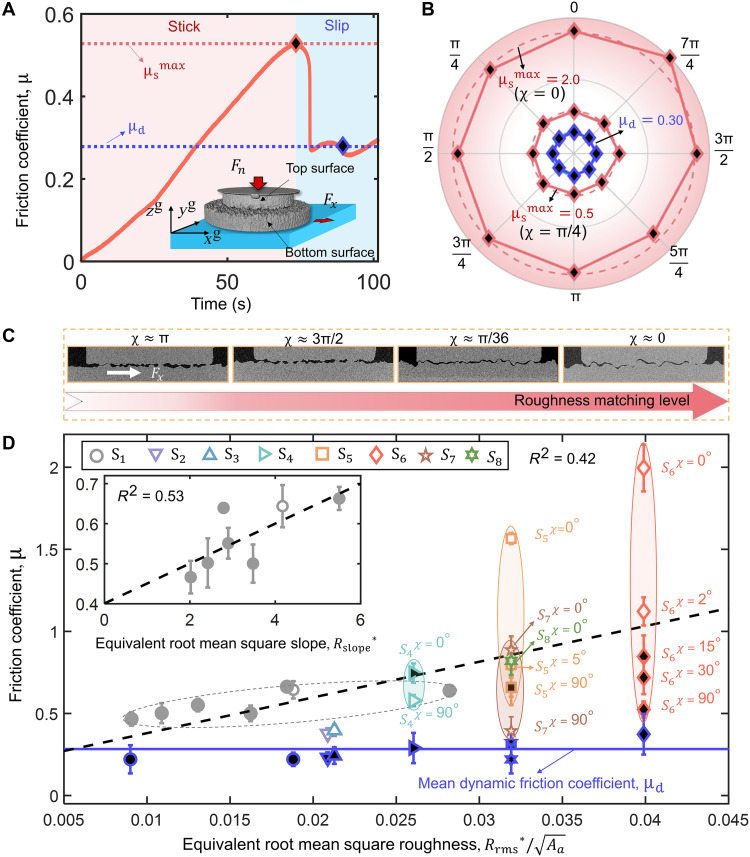
Friction at contact pairs with different roughness alignments. (**A**) Friction evolution for a typical rough-rough contact. The red and blue regions represent the static and dynamic friction regimes, respectively, with the red and blue dashed lines (marked with diamonds) indicating the maximum static and dynamic friction coefficients. The inset shows the sketch of the rough-rough contact under shear. (**B**) Static (red diamond) and dynamic (blue diamond) friction coefficients measured along different shear directions for sample $S_{6}$ . The matching angle χ = 0 corresponds to optimal mating alignment, while χ = π /4 represents a lower roughness matching level. The dotted line represents the average value of the maximum static friction coefficient measured at different shear directions. (**C**) Contact configurations with different roughness matching levels tuned by rotating the top contacting surface. (**D**) The relationship between the maximum static friction coefficient, ${\mu_{s}}^{\text{max}}$ , and the dimensionless equivalent root mean square roughness ${R_{\text{rms}}}^{*}/\sqrt{A_{a}}$ for different contact pairs. Here, $A_{a}$ is the apparent contact area. The solid blue line is the mean coefficient of dynamic friction $\mu_{d}$ . The black dashed line is the linear fit to all tested contact pairs, with the determination coefficient of around 0.42. The gray solid circles show the contact between the flat surface and surfaces with different $R_{\text{rms}}$ values, and the insert shows the dependence of ${\mu_{s}}^{\text{max}}$ on the equivalent root mean square slope ${R_{\text{slope}}}^{*}$ for different rough-flat contacts. Markers filled with white represent results with the in situ 3DXRCT measurements. Present average values and SDs are obtained over five measurements. *R*^2^, coefficient of determination.

We conduct stick-slip friction tests on contact pairs with varying random roughness, as shown in Fig. 1A. Three groups of rough contacts are considered, each differing in contact type and roughness matching degree, which is preliminarily estimated on the basis of initial interfacial porosity (fig. S1D): (i) the flat-to-rough contact, i.e., $S_{1}$ ; (ii) randomly rough-to-rough contacts with relatively large interfacial porosity, represented by $S_{2}$ , $S_{3}$ , and $S_{4}$ ; and (iii) well-mated rough-to-rough contacts, including $S_{5}$ , $S_{6}$ , $S_{7}$ , and $S_{8}$ , whose roughness matching level can be adjusted by rotating the top rough surface by a certain angle, defined as the roughness matching angle, χ . In particular, our experiments examine two representative types of rough surfaces: Gaussian surfaces ( $S_{1}\;\text{to}\;S_{6}$ ) with normally distributed heights and self-affine fractal surfaces ( $S_{7}$ and $S_{8}$ ). These morphologies comprehensively capture the characteristic roughness features of both natural and engineering surfaces commonly found in real-world applications (*16*, *18*, *42*). Further details on the preparation, characterization, and experiments for the rough contacts are provided in Materials and Methods and table S1. For the well-mated rough-rough contacts with a high matching level, i.e., $S_{6}$ , the maximum static friction coefficient ${\mu_{s}}^{\text{max}}$ , which reflects the static frictional strength, is observed to be substantially higher than the dynamic friction coefficient $\mu_{d}$ , as shown in Fig. 1D. Here, ${\mu_{s}}^{\text{max}}$ peaks with the matching angle χ=0° corresponding to a perfect roughness matching state. As the matching angle deviates from this optimal alignment due to relative rotation between the top and bottom surfaces, ${\mu_{s}}^{\text{max}}$ decreases. In contrast, the dynamic friction coefficient $\mu_{d}$ remains stable over varying matching angles, consistent with previous studies (*43*, *44*). Note that the surfaces considered in this study are randomly rough and geometrically isotropic, resulting in evenly distributed asperity interlocking. For a fixed mating alignment, the measured friction coefficients exhibit minimal directional variation (see Fig. 1B), confirming that the frictional response remains isotropic. By comparing ${\mu_{s}}^{\text{max}}$ across all tested contacts with various roughnesses, we observe a slight increase in ${\mu_{s}}^{\text{max}}$ as a function of $R_{\text{rms}}^{*}=\sqrt{{R_{\text{rms}}^{T}}^{2}+{R_{\text{rms}}^{B}}^{2}}$ , with $R_{\text{rms}}^{T}$ and $R_{\text{rms}}^{B}$ being, respectively, the root mean square roughness of the top and bottom surfaces (*45*), as indicated by the dashed line in Fig. 1D When restricting the analysis to rough-flat contacts with varying equivalent root mean square slope, $R_{\text{slope}}^{*}=\sqrt{{R_{\text{slope}}^{T}}^{2}+{R_{\text{slope}}^{B}}^{2}}$ , given by the root mean square slope of the top and bottom surfaces, i.e., $R_{\text{slope}}^{T}$ and $R_{\text{slope}}^{B}$ , respectively, an increasing trend is also observed in the insert of Fig. 1D, consistent with previous studies (*19*, *23*). However, results in green, brown, orange, and red markers for contacts with varying roughness matching levels suggest that it is insufficient to predict the ${\mu_{s}}^{\text{max}}$ solely based on roughness parameters. The roughness matching level can notably influence ${\mu_{s}}^{\text{max}}$ over a wide range. This dependence arises from the degree of asperity interlocking, which is governed by the geometric complementarity of the surfaces at different matching angles. By precisely controlling the matching angle χ , we can continuously adjust the roughness alignment level and, therefore, the contact conformability. For a given contact pair with fixed roughness matching, friction remains isotropic due to the statistical isotropy of both surfaces. Rotating the surfaces alters the matching level, uniformly changing frictional strength in all directions.

### Universality in distributions of micromechanical parameters

To uncover the underlying mechanics of the stick-slip friction at rough contacts with different roughness matching levels, we perform in situ 3DXRCT measurements (detailed in Materials and Methods) to analyze contact evolution under successive shear loading steps. We then extract the overall contact parameters and micromechanical quantities for microcontacts across load steps. We observe that the rough-flat contact $S_{1}$ and highly matched contact, such as $S_{6}$ , exhibit comparable microcontact mechanisms, including contact area evolution, porosity changes, and contact deformation during shear (see fig. S1). These behaviors contrast with those observed in rough-rough contacts with lower matching levels, such as $S_{2}$ , $S_{3}$ , and $S_{4}$ . Specifically, under the same shear loading procedures, contact areas of $S_{1}$ and $S_{6}$ increase by about 50%, whereas those of $S_{2}$ , $S_{3}$ , and $S_{4}$ fluctuate within 10%. Values of interfacial porosity of $S_{1}$ and $S_{6}$ reduce by up to 25%, while those of $S_{2}$ , $S_{3}$ , and $S_{4}$ vary by less than 10%. Notably, $S_{6}$ exhibits the highest $R_{\text{rms}}^{*}$ among all samples, while the rough-to-flat contact $S_{1}$ has the lowest $R_{\text{rms}}^{*}$ (table S1). The comparable properties observed between contacts with the lowest and highest roughness, i.e., $S_{1}$ and $S_{6}$ , respectively, suggest that the previously reported monotonic relationships (*18*, *24*, *46*) between contact area, interfacial separation, and porosity may not hold for rough-rough contacts, where roughness matching plays an important role.

**Fig. 2.**
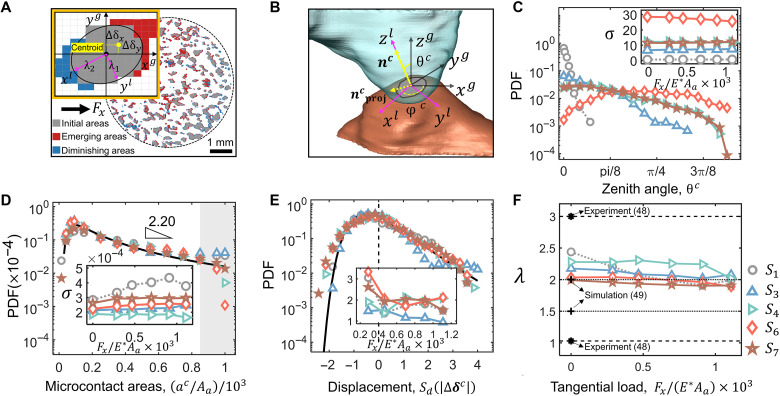
Statistical distributions of micromechanical quantities. (**A**) Top view of contact topography for $S_{1}$ under the normal compression of 83.06 N and the tangential force of 25.20 N. The left corner is a top view of the evolution of a typical microcontact under shear. The black and yellow dots denote, respectively, the initial and updated centroids of the microcontact. $∆\delta_{x}$ and $∆\delta_{y}$ represent the displacements of the microcontact centroid along x and y axes, respectively. The microcontact centroid is determined through the ellipsoid fitting using all XRCT pixels constructing the corresponding microcontact. The aspect ratio of the microcontact area is defined as the ratio between the lengths of the two largest principal semi-axes of the fitted ellipsoid, $\lambda_{1}/\lambda_{2}$ . (**B**) The 3D diagram for a typical oblique microcontact between two asperities. Here, $\theta^{c}$ is the zenith angle, i.e., the angle between $n^{c}$ (the unit vector perpendicular to the microcontact plane) and ${n_{z}}^{G}$ (the unit vector along $z^{g}$-axis direction). (**C** and **D**) Probability density distributions, respectively, for the zenith angle and microcontact area extracted at the first load step with only normal compression. The inserts in (C) and (D) show the corresponding scale parameter σ across load steps. Gray shaded region indicates the largest microcontact areas of PDFs. (**E**) Probability distributions of relative microcontact deformation with respect to the previous loading step, calculated by the displacement of microcontact centroid, i.e., $|\;∆\delta^{c}\;|\;=|\;{\left(∆\delta_{x},∆\delta_{y},∆\delta_{z}\right)}^{g}\;|$ . The black lines in (D) and (E) fit the probability density curve of GEV for all samples, with the goodness of fitting higher than 0.95. The respective mean values of $|\;∆\delta^{c}\;|$ over microcontacts at each load step are presented in the insert of (E). (**F**) The decay exponent λ of the power-law distribution varies with the shear load.

We extract micromechanical quantities of each individual microcontact across load steps, including orientation, size, shape, and displacement. The probability distribution functions (PDFs) for the zenith angle of microcontact, $\theta^{c}$ , microcontact area, $a^{c}$ , and displacement $|\;∆\delta^{c}\;|$ extracted at the initial load step under pure normal compression are compared for different samples in Fig. 2, C to E, respectively. Here, to reasonably combine datasets from different loading steps and across all contacts, the data collected for the contact at a given measurement step are normalized with the standard score, i.e., $S_{d}\left(X_{i}\right)=\left(X_{i}-\bar{X}\right)/\text{std}\;\left(X\right)$ . The X represents the dataset, and $\bar{X}$ and std(X) are the mean and SD, respectively. Additional comparisons of PDFs of micromechanical quantities across shear load steps are provided in fig. S2. Notably, for each micromechanical quantity, PDFs across all load steps exhibit identical trend and shape, as further supported by relatively stable Gini coefficients (see fig. S3). Except for the zenith angle of microcontact shown in Fig. 2B, the PDFs of all other micromechanical quantities collapse onto a single curve across different samples, exhibiting consistent distributions for both Gaussian and fractal surface contacts. This suggests a common statistical pattern for micromechanical quantities across all contact pairs, irrespective of surface roughness, roughness matching degree, shear load, and direction. To quantify this observed universality, we compare several statistical models commonly used in contact mechanics to fit the extracted distributions of micromechanical quantities. Among these candidates, the generalized extreme value (GEV) distribution consistently provided the best fit across all tested variables and load steps. The results are supported by relative Akaike weights, which account for both goodness of fit and model complexity, as shown in figs. S4 to S7. The PDFs of GEV distribution can be described as follows (*4*)

ϕ(x)=1σt(x)k+1e−t(x) (1)

where $t\left(x\right)={\left[1+k\left(x-\mu \right)/\sigma \right]}^{-1/k}$ with x as the variable, k being the shape parameter, and μ and σ representing the location and scale parameters, respectively, which can be approximated by the mean and the SD of x . The GEV distribution is a unified model that encompasses three types of extreme value behaviors (Gumbel, Fréchet, and Weibull), making it flexible for capturing the skewed, heavy-tailed, and non-Gaussian features that emerge in our micromechanical datasets. This is particularly relevant in randomly rough surfaces, where the interaction among asperities leads to emergent phenomena, such as interlocking, localized yielding, and intermittent slip. Moreover, we also extract GEV parameters, including k , μ , and σ , for all tested contact pairs by fitting experimental data and compare these values in tables S2 to S20. The value of k for microcontact areas is found to increase slightly with the applied shear load (see fig. S8), indicating that the distribution of $a^{c}$ continues to expand as shear increases (*31*). However, this variation is mainly driven by the limited number of the largest microcontacts, as shown by the gray zone in Fig. 2D, while most PDFs retain their universality. The power-law decay distributions can be derived from the obtained GEV functions, i.e., $\phi \left(x\right)\propto x^{-\lambda }$ , where the decay exponent is calculated as λ=(k+1)/k (detailed in the Supplementary Materials). The obtained values of λ in our experiments range from about 1.85 to 2.4, comparable to previously reported values (*47*, *48*) for rough contacts under various normal compressions, as shown in Fig. 2F. Regarding the exception to the observed universal statistical behavior, while distributions of zenith angle $\theta^{c}$ for a given contact pair under different shear loadings remain similar, disparities arise for different contact pairs in terms of spread width (Fig. 2C, insert), mean value (tables S2 to S8), and symmetry (Fig. 2C). Intuitively, the microcontact zenith angle, $\theta^{c}$ , serves as a direct indicator of asperity interaction in rough-rough contacts with varying roughness matching levels. The remaining challenge is how to comprehensively incorporate the orientation characteristics of all microcontacts into a single, physically meaningful, and well-defined parameter.

### Quantifying roughness matching with contact fabric tensor

To quantitatively describe interfacial roughness matching, we implement the concept of fabric tensor to microcontact collectives (*49*). The contact fabric tensor F is computed by summing the unit normal vector $n^{c}$ and its opposite vector $-\;n^{c}$ for each microcontact, as detailed in Materials and Methods. The component of the obtained symmetric tensor F is defined as

**Fig. 3.**
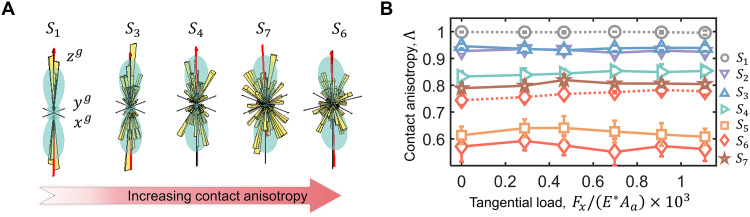
Evolution of contact anisotropy. (**A**) 3D rose diagram of distributions for microcontact orientations of different contact pairs under the tangential load of 20.70 N. The yellow bars represent the discrete probability distribution of microcontact normal vectors, and the cyan maps represent the continuous probability distribution $Ε\left(n^{c}\right)$ . The solid black lines represent the global 3D coordinates, and the solid red arrow pointing upward is the maximum principal direction of the contact fabric tensor. (**B**) The dependence of contact anisotropic index on the tangential load for contacts with various roughness matching levels. The reported average values and their corresponding SDs are obtained based on five XRCT measurements.

Fij=∫ΩninjdΩ=1N∑1Nninj,(i,j=1,2,3) (2)

where i and j mean orthogonal components, Ω is the solid angle for the entire surface and equals 4 π in 3D space, and N is the number of microcontacts. On the basis of the obtained contact fabric tensor, we calculate the eigenvalues and eigenvectors, i.e., $F_{i}$ and $V_{i}$ ( i= 1, 2, and 3), where $F_{1}$ and $F_{3}$ are the smallest and largest eigenvalues, respectively, corresponding to the eigenvectors $V_{1}$ and $V_{3}$ . Then, the roughness matching level for a rough contact is characterized by the contact anisotropic index, $\Lambda =\left(F_{3}-F_{1}\right)/\left(F_{3}+F_{1}\right)$ . To interpret the micromechanical meaning of Λ , we visualize the distribution of microcontact normal vectors $n^{c}$ in 3D rose diagrams, together with the fitted probability density function $Ε\left(n^{c}\right)$ . The continuous probability density function is further expressed using a second-order spherical harmonic expansion, from which the contact anisotropy index can be analytically estimated. Details are provided in Materials and Methods. Because of the inherent symmetry of the fabric tensor, the 3D rose diagram exhibits a symmetrical dumbbell shape, as shown in Fig. 3A. We find that the $n^{c}$ of rough-flat contact $S_{1}$ in microcontacts are nearly perpendicular to the flat surface and Λ approaches 1 (Fig. 3B). As the roughness matching enhances, the rose diagram continues to expand laterally, accompanied by the shifting away of microcontact normal vectors from the $z^{g}$ axis (fig. S10). The increasing presence of tilted microcontacts leads to a reduction in Λ , indicating a higher proportion of shoulder-to-shoulder asperity interactions. The value of the defined contact anisotropic index Λ ranges from 0 to 1, thereby offering a valuable metric directly applicable to predictive models for assessing roughness matching and its impact on frictional behavior.

### The dependence of friction strength on roughness alignment

**Fig. 4.**
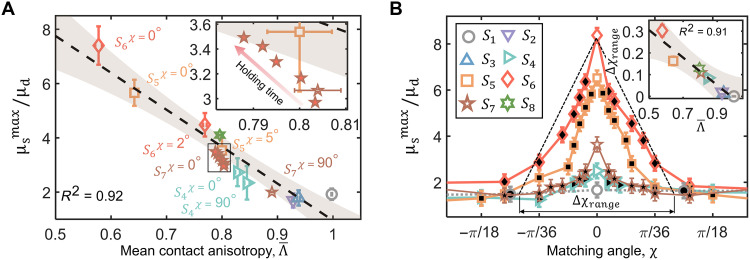
Relationship between friction coefficient and roughness matching level. (**A**) The dependence of ${\mu_{s}}^{\text{max}}/\mu_{d}$ on the mean contact anisotropy. The black dashed line represents a linear fit, while the gray shaded area indicates the 95% confidence interval. The red-filled pentagrams represent the evolution of frictional behavior for $S_{7}$ , subjected to different holding durations under normal compression of 83.06 N. (**B**) The dependence of ${\mu_{s}}^{\text{max}}/\mu_{d}$ on the roughness matching angle χ . The marker filled with white indicates results with in situ 3DXRCT measurements. The insert shows the dependence of the tuning range ${\Delta \chi }_{\text{range}}$ on the mean contact anisotropy $\bar{\Lambda }$ . Here, the reported average values and their corresponding SDs are calculated over five measurements.

The computed contact anisotropy Λ for all tested contacts remains relatively stable across load steps before transitioning into the fully sliding regime (Fig. 3B). This indicates the contact fabric tensor’s ability to capture the universality of the distribution of microcontact orientations throughout the shear process, even as the stick-slip transition involves complex phenomena such as slipping, elastoplastic deformation, material yielding, and abrupt fractures of small microcontacts (*13*). Further correlation analysis reveals a strong linear negative relationship between the ratio of ${\mu_{s}}^{\text{max}}$ to $\mu_{d}$ and the contact anisotropic index Λ , exhibiting the determination coefficient of around 0.92, as shown in Fig. 4A. This observed linear correlation underscores the mechanical significance of interfacial microstructures: As the contact anisotropy decreases, microcontacts tilt more, enhancing asperity interlocking and increasing the energy required to initiate slip. Consequently, interfaces with lower contact anisotropy consistently exhibit greater maximum static friction. This trend also appears during interfacial aging, where the prolonged holding time leads to a reduced contact anisotropy and increased ${\mu_{s}}^{\text{max}}$ as illustrated in the insert of Fig. 4A. The state dependence of ${\mu_{s}}^{\text{max}}$ is quantitatively incorporated into the predictive trend via the contact fabric tensor. In contrast, no obvious correlation is observed between $\mu_{d}$ and Λ , as $\mu_{d}$ remains nearly stable across all tested samples under the same loading condition (Fig. 1D).

These results provide a micromechanical interpretation of macroscopic static friction and establish a predictive framework linking interface-scale roughness to bulk frictional behavior. To further elucidate this linkage, we propose a simplified model that relates the maximum static friction coefficient to the microcontact orientation through the fabric tensor. As detailed in Materials and Methods, the model predicts a negative linear relationship between the contact anisotropy index and the maximum static friction coefficient. Theoretical calculations for this linear relationship based on Eq. 13 yield a slope of approximately 1.47, which captures the correct trend but underestimates the magnitude relative to the slope of 2.69 obtained from linear regression with experimental data (Fig. 4A). This discrepancy may stem from the model’s assumption of idealized planar contacts extracted from CT scans and its omission of microcontact force magnitudes and their fluctuations, which warrant further investigation.

By adjusting the matching angle χ for the highly matched contact $S_{6}$ , we find that the ${\mu_{s}}^{\text{max}}$ can be tuned between $\mu_{d}$ and up to more than eight times of $\mu_{d}$ , as shown in Fig. 4B. This substantial variation of ${\mu_{s}}^{\text{max}}$ further shows that contacts with higher levels of roughness matching lead to increased static frictional strength, driven by greater interfacial potential energy accumulation due to asperity interlocking (*50*). However, this frictional enhancement is suppressed in rough contacts with lower matching levels and rough-flat contacts. We further quantify the tunable range of roughness matching angle, $∆\chi_{\text{range}}$ , which represents the adjustable range of interfacial strength. This is done by identifying the corresponding matching angle at which the ${\mu_{s}}^{\text{max}}$ decays from its peak value to the mean level, as illustrated in Fig. 4B. Results show that $∆\chi_{\text{range}}$ is a strong correlation with both the contact anisotropy index Λ and macroscopic roughness parameters, such as $R_{\text{rms}}$ (fig. S12), with a coefficient of determination exceeding 0.90 (Fig. 4B, insert).

## DISCUSSION

The multiscale roughness inherent in both natural and engineered surfaces plays a dominant role in determining complex interfacial properties across different length scales. Despite its importance, challenges persist in understanding the frictional behavior of randomly rough contacts. In our experiments, we observe that the highly matched rough-rough contact, $S_{6}$ , exhibits a remarkably higher static frictional strength, compared with that of the rough-flat contact, $S_{1}$ . Through in situ 3DXRCT measurements, we find that the two types of contacts demonstrate comparable macroscopic properties in terms of contact area, contact deformation, interfacial porosity, and their evolutions under shear, although the former presents evidently larger equivalent root mean square roughness and slope. This challenges widely accepted monotonic functions between contact properties and roughness parameters (*2*, *16*, *24*, *51*, *52*), emphasizing the necessity of considering the roughness matching level in studying rough-rough contacts.

In addition, when incorporating our experimental observations with the rate-and-state friction (RSF) framework (*34*), the roughness matching level substantially influences the evolution of the state variable and frictional response of the stick-slip transition. The steady-state friction coefficient can be described by the RSF theory (*53*) as $\mu =\mu_{*}+\left(a-b\right)\text{ln}\left(V/V_{*}\right)$ , where $\mu_{*}$ is the friction coefficient at a reference velocity $V_{*}$ (here $V_{*}=5\;\mathrm{\mu m}/s$ ). As the sliding velocity increases, the interface exhibits velocity-weakening behavior across different roughness matching levels, as indicated by the negative slope values of (a−b) shown in fig. S13. The slope values for contacts of different matching levels are quite close, i.e., approximately 0.012, suggesting a minimal influence of the initial contact configuration on the steady-state friction. However, the roughness matching degree substantially affects the stick-slip transition. As the sliding velocity increases, the maximum static friction coefficient, ${\mu_{s}}^{\text{max}}$ , exhibits a weakening trend. This rate-induced reduction in ${\mu_{s}}^{\text{max}}$ is more pronounced at higher roughness matching levels, as shown in the inset of fig. S13B. While stronger asperity interlocking typically enhances static friction, increased shear velocities lead to a quicker degradation of this interlocking, suggesting that the contact becomes less resistant to slip as the velocity increases. This phenomenon highlights the velocity-dependent nature of frictional strength, especially in configurations with high geometric conformity.

To investigate the influence of the state variable within the RSF framework, we perform aging friction tests combined with in situ 3DXRCT experiments to capture the evolution of the interfacial structure under different holding times and examine the influence of contact aging on frictional behavior. According to previous research (*53*–*56*), the aging law for the friction coefficient can be described by ${\mu_{s}}^{\text{max}}={\mu_{*}}^{\text{max}}+\mu_{\beta }\text{ln}\left(1+t/t_{*}\right)$ , where ${\mu_{*}}^{\text{max}}$ and $\mu_{\beta }$ are experimental constants, and $t_{*}$ is a reference time. By fitting the obtained experimental data with the equation above, we observe that the contact with a higher roughness matching level shows a more pronounced increase in ${\mu_{s}}^{\text{max}}$ (see fig. S14), indicating enhanced interfacial stability with stronger asperity interlocking. The extracted contact anisotropy index Λ based on 3DXRCT measurements is found to negatively correlate with the measured ${\mu_{s}}^{\text{max}}$ , and this correlation can be well integrated into datasets present in Fig. 4A. This result demonstrates that both the time-evolved interfacial structure and the variation of roughness matching can be effectively captured by the proposed framework based on the contact fabric tensor.

Our experimental observation on universal patterns highlights the effectiveness and accuracy of GEV distributions in describing the statistical properties of micromechanical quantities and their evolution at isotropic rough contacts during shear. Key micromechanical quantities, including microcontact area, shape, displacement, and orientation, are found to follow GEV distributions. For each micromechanical quantity (excluding microcontact zenith angle), obtained distributions during shear can be collapsed onto a universal curve, independent of contact types (rough-flat or rough-rough contacts), surface morphology (Gaussian or fractal surfaces), the applied shear load, and direction. The observed universality partially supports the common practice of simplifying rough-rough contacts as rough-flat contacts. The exception to this universality arises in the distributions of microcontact zenith angles across different contact pairs, demonstrating different levels of roughness matching. Previous studies have analytically and numerically explored microcontact orientations to explain frictional resistance and shear displacement hardening in rough-rough contacts (*26*, *57*, *58*). These models typically assume spherical and ellipsoidal asperities with simple, symmetric distributions of microcontact orientations, such as Gaussian. Our in situ XRCT quantifications reveal that this distribution of microcontacts at randomly rough contacts is non-Gaussian and closely approximates the GEV function. This finding provides valuable insights for refining micromechanics-based models and advancing predictive models of frictional behavior in rough contacts. Moreover, the exception to the observed universality underscores the potential for a quantitative method to distinguish rough-rough contacts based on their roughness matching levels. We propose the use of the contact fabric tensor to account for the effects of microcontact orientations. The contact anisotropy, derived from the contact fabric tensor, serves as an experimentally measurable index ranging from 0 to 1, that is capable of quantifying the roughness alignment levels. The computed contact anisotropy is strongly correlated with the maximum friction coefficient at the stick-slip transition, providing a direct measure of static frictional strength.

In conclusion, the introduction of the GEV function and contact fabric tensor, respectively, summarizes and then unifies micromechanical statistics at isotropically rough contacts and enhances both theoretical and experimental understanding of how roughness matching influences frictional behavior across stick-slip and sliding regimes. This study also highlights a promising strategy that, by precisely controlling roughness features and alignment at the mesoscopic scale, frictional properties can be finely tuned and optimized for enhancing performance, offering insights into applications ranging from mechanical systems and bioinspired adhesives to earthquake modeling.

## MATERIALS AND METHODS

### Sample preparation

Two representative types of randomly rough surfaces widely studied in contact mechanics are considered: Gaussian rough surfaces with normally distributed roughness heights and multiscale fractal surfaces exhibiting scale-invariant features. The isotropic Gaussian surfaces are simulated on the basis of a fast Fourier transform algorithm with two roughness parameters controlled (*59*), i.e., root mean square roughness $R_{\text{rms}}$ and autocorrelation length $\beta_{0}$ . The surface height follows a Gaussian distribution, and the autocorrelation function decays exponentially. The fractal rough surfaces are generated using a standard Fourier filtering method (*60*), where the surface height is constructed to follow a power-law form of the power spectral density, as described by eq. S1 in the Supplementary Materials. The fractal dimension is set to $D_{f}$ = 2.15 to reflect realistic surface morphologies (*42*). More details are provided in section S2 and table S1 of the Supplementary Materials. The simulated rough surfaces are then 3D-printed by ProJet MJP 3600max with a spatial resolution of about 10 μm, ensuring high fidelity in reproducing the designed morphologies. The material used for 3D printing is VisiJet Crystal, an ultraviolet-curable plastic, whose modulus of elasticity E is 1.463 GPa, Poisson ratio ν is 0.3, and yield strength $\sigma_{Y}$ is 42.4 MPa. The diameters of the top and bottom specimens are 6 and 9 mm, respectively, ensuring that the top rough surface is fully contained within the bottom surface. A substrate of 1 mm in thickness is included within the printing process below the valley of the rough surface to maintain the overall flatness and increase resistance to bending. This fabrication approach enables the production of a sufficient number of geometrically consistent, high-quality contact pairs for each surface type. Before experiments, all samples are properly cleaned using ultrasonic cleaning with an ethanol-based detergent to ensure the removal of residues and contaminants.

### Friction experiments

Different levels of roughness matching at the contact pair are accomplished using marker structures patterned on both the top and bottom surfaces (fig. S15). For well-mated rough-to-rough contacts, including $S_{5}$ , $S_{6}$ , $S_{7}$ , and $S_{8}$ , the degree of alignment can be precisely tuned by adjusting the matching angle χ . The perfect alignment ( χ=0 ) is readily attainable due to geometric complementarity, as the bottom surface is a negative replica of the top surface. All other matching angles are calibrated relative to this reference configuration. The target matching angle is then achieved by rotating a high-precision torsional motor, which is rigidly mounted to the upper surface holder (fig. S16). The exact value of χ is quantified by measuring the relative angle between the central lines of top and bottom markers, using either 3DXRCT imaging or photographic analysis during friction testing (fig. S15). On the basis of the self-developed testing system shown in fig. S16, we conduct a series of slow friction tests at a sliding speed of 5 μm/s , maintaining a constant normal contact deformation. The normal contact deformation is initially applied by the normal compression of 83 N (consistent with the normal load maintained during in situ 3DXRCT tests) and is subsequently maintained throughout the test. The normal contact force and frictional force are recorded by a triaxial force sensor with a resolution of 0.05 N. During the sliding process, we record the evolution of the contact with an optical camera at a framerate of 40 Hz. On the basis of these images, the normal and tangential contact deformation are extracted using the normalized cross-correlation algorithm (*61*). To eliminate the effects of interface fracture, wear, and material plasticity, a new contact pair is used for each friction test. To ensure statistical robustness and repeatability, each friction test in this study is repeated five times under identical alignment and loading conditions.

### Procedure of 3DXRCT experiments

Apart from friction tests, in situ 3DXRCT tests are conducted for contacts under constant normal compression load and incrementally increasing tangential loads, via a custom-built loading device within the scanning chamber of the computed tomography (CT) scanner (ZEISS Xradia 610). In the experiment, the top and bottom surfaces are glued on their respective top and bottom sample holders. The top sample holder can only translate vertically along the central axis of the device, constrained by a linear guide. The relative position of the bottom sample holder can be adjusted horizontally relative to the fixed base using a linear slider. The bottom holder is further connected to the side dead weight via a nylon rope wrapped around a rolling bearing, allowing the gravitational force of the side dead weight to be converted into horizontal force, thereby applying tangential force to the contact sample. During the experiment, the normal load, $F_{n}=83.06\pm 0.10\;N$ , is maintained by the combined weight of components attached to the top holder, including the top dead weight, dead weight holder, linear guide, and top sample holder. Across six successive measurement steps, the tangential force increases from 0 to 25.20 N in increments of ~5 N by adding side dead weights while ensuring that the testing contact remains within the static friction regime. During each XRCT scan, the entire contact is illuminated by a 120-keV x-ray beam, with each CT scan lasting less than 20 min and achieving a spatial resolution of ~7.6 μm.

### Extracting contact evolutions

**Fig. 5.**
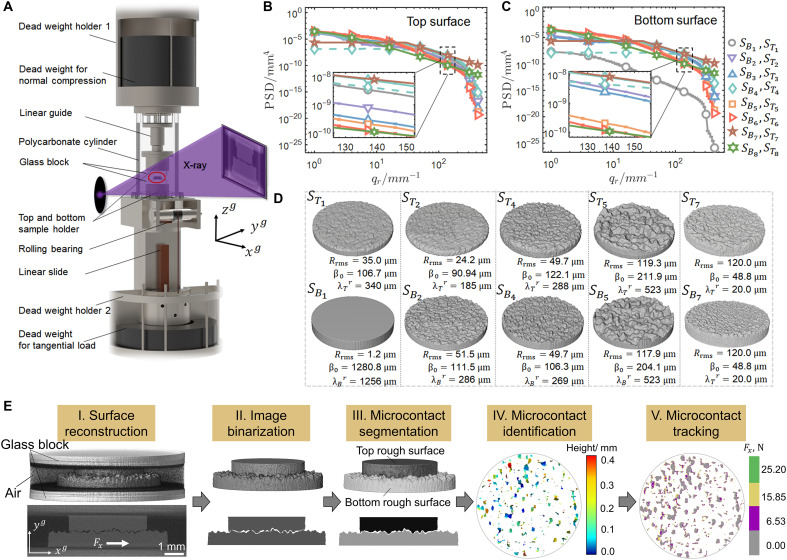
Experimental setup and rough contacts. (**A**) Schematic of the self-developed loading system for in situ 3DXRCT measurements. The position of the red circle is the contact pair under test. (**B** and **C**) 2D power spectral densities (PSD), respectively, for top and bottom surfaces of contact pairs. The inserts provide a magnified view of the dotted rectangular box. The cyan diamond dashed line represents the power spectrum of the small perturbations superimposed on sample $S_{4}$ . (**D**) Reconstructed 3D contact structures under the normal compression of $F_{n}$ . The first (second) row is the top (bottom) 3D rough surface. $R_{\text{rms}}$ and $\beta_{0}$ are calculated by the reconstructed surface structures. Note that the top rough surface is magnified by 1.5 times in equal proportions, so it is visually the same size as the bottom rough surface. (**E**) Image processing flow of a typical sample $S_{5}$ . The main steps include reconstruction, binarization, segmentation, microcontact identification, and tracking.

The contact structure under normal compression and its evolution across shear load steps are extracted from obtained 3DXRCT images. Image processing is performed using self-developed algorithms in sequential steps: reconstruction, binarization, segmentation, and microcontact identification and tracking (Fig. 5E) (*62*). First, the Otsu’s algorithm (*63*) is used to globally threshold 3D images, followed by a local interactive thresholding procedure to identify voids and solids. Binarization is then performed, followed by morphological opening and closing procedures with the 3D window of 3×3×3 pixels, to enhance the signal-to-noise ratio. Subsequently, the top and bottom surfaces are segmented using the watershed algorithm (*64*). The pixels representing the real contact are identified through the contact search algorithm (*62*). Individual microcontacts at the contact interface are further segmented, identified, labeled, and tracked over load steps.

### Estimating the maximum static friction coefficient

The local coordinate ( $o^{L}x^{L}y^{L}z^{L}$ ) is first introduced for each individual microcontact (Fig. 2B). These local coordinates are determined using the singular value decomposition algorithm (*65*). For each microcontact, the $z^{L}$ axis of the local coordinate system is perpendicular to the fitted plane of the microcontact area, and the $x^{L}$ and $y^{L}$ axes correspond to the directions of the maximum and minimum eigenvectors of the microcontact area, respectively. Through the Euler rotation, represented by $R_{\mathrm{ZYX}}\left(\alpha ,\beta ,\gamma \right)$ , characteristics of microcontacts in their respective local coordinates can be then transformed into the global coordinate system, as detailed in section S3 of the Supplementary Materials. The probability density distributions of Euler angles (α,β,γ) for all microcontacts across load steps are provided in fig. S9. With the rotation matrix linking local and global coordinate systems, the zenith angle $\theta^{c}$ and azimuth angle $\varphi^{c}$ for each microcontact with respect to the global coordinate system can be then calculated to describe microcontact orientation. In addition to these angles, the microcontact orientation can be further quantified by the corresponding normal unit vector, defined as $n^{c}={\left(\;\text{cos}\theta^{c},\text{cos}\varphi^{c}\text{sin}\theta^{c},\text{sin}\varphi^{c}\text{sin}\theta^{c}\right)}^{T}$ . For visualizing the spatial distributions of the microcontact orientation, we generate a smoothed continuum PDF for $n^{c}$ , through the 3D rose diagram. Different types of approximations of the probability density function $Ε\left(n^{c}\right)$ have been proposed (*66*). Here, we adopt the second-order truncation of the Fourier expansion of the orientation distribution function

Ε(nc)=12π(1+Dijninj),(i,j=1,2,3) (3)

where $D_{\mathrm{ij}}=15/2\left(F_{\mathrm{ij}}-\delta_{\mathrm{ij}}/3\right)$ is the second-order deviatoric fabric tensor, and $\delta_{\mathrm{ij}}$ is the Kronecker delta. For an interface with an isotropic distribution of asperity contact orientations, the coefficient tensor $D_{\mathrm{ij}}$ is given by (*67*)

[Dij]=[a20000−a202+3a223b2203b22−a202−3a22] (4)

Here, $a_{20}$ , $a_{22}$ , and $b_{22}$ are constants determined experimentally based on the components of the fabric tensor $F_{\mathrm{ij}}$ and are assoiciated with the distribution of microcontact orientation (*68*). Substituting Eq. 4 into Eq. 3 and using $n^{c}={\left(\;\text{cos}\theta^{c},\text{cos}\varphi^{c}\text{sin}\theta^{c},\text{sin}\varphi^{c}\text{sin}\theta^{c}\right)}^{T}$ , the 3D probability density function is given as

Ε(θ,φ)=12π[1+14a20(3cos2θc+1)+3sin2θc(a22cos2φc+b22sin2φc)] (5)

Therefore, the shape of this probability density function is governed by the parameters $a_{20}$ , $a_{22}$ , and $b_{22}$ . The eigenvalues of $D_{\mathrm{ij}}$ can be calculated as

D1,2=−a202±3a222+b222 (6)

D3=a20 (7)

With the relationship between the coefficient tensor $D_{\mathrm{ij}}$ and the fabric tensor $F_{\mathrm{ij}}$ , the eigenvalues of the fabric tensor can be determined by

Fi=215λiD+13 (8)

and the contact anisotropic index is given by

Λ=32a20−3a222+b22212a20+3a222+b222 (9)

In dry friction, the frictional force is primarily governed by surface adhesion (e.g., van der Waals forces) and surface roughness (*69*). Given the weak surface adhesion of the materials used in this study (*28*), the interfacial friction force is mainly controlled by the surface roughness. To estimate the macroscopic friction coefficient, we assume that each microscopic oblique contact adheres to coulomb’s friction law (*70*). The local friction coefficient $\mu_{\text{local}}$ is determined by the ratio of local shear force and normal stress, i.e., $\mu_{\text{local}}=f_{x}/f_{n}$ , where $f_{x}$ and $f_{n}$ are the tangential and normal forces during sliding. Notably, the $\mu_{\text{local}}$ is related to the material properties, and the average value of $\mu_{\text{local}}=$ 0.3 ± 0.04 is adopted over five meansurements. On the basis of the previous research (*44*), the microscopic friction coefficient $\mu_{\text{micro}}$ is determined by the $\mu_{\text{local}}$ and the microcontact orientation, i.e.

μmicro=tan(θc+tan−1μlocal) (10)

By combining Eq. 5, which describes the probability density distribution of microcontact orientations, we can estimate the macroscopic maximum friction coefficient as

μsmax~∫0θmax∫02πΕ(θc,φc)μmicrosinθcdθcdφc (11)

where $\theta_{\text{max}}$ is the maximum zenith angle of microcontacts, which depends on the roughness matching levels. Evaluating the integral in Eq. 11, an estimate of the maximum static friction coefficient is obtained

μsmax~[μlocal+ln(1cosθcmax+tanθcmax)−sinθcmax+a202(sinθcmax+sin3θcmax−ln(1cosθcmax+tanθcmax)+3μlocal2)] (12)

This equation indicates that the maximum static friction coefficient is influenced by both the roughness matching and material properties. By combining Eq. 12, the linear relationship between the maximum static friction coefficient ${\mu_{s}}^{\text{max}}$ and the contact anisotropy index Λ can be established

μsmax~−Η(θcmax,μmicro,a22,b22)Λ (13)

Here, $\begin{array}{c} H\;\left({\theta^{c}}_{\text{max}},\mu_{\text{micro}},\;a_{22},\;b_{22}\right)\;=\;4/3\;\sqrt{{a_{22}}^{2}+{b_{22}}^{2}}\;\left(\text{sin}{\theta^{c}}_{\text{max}}+\right. \end{array}$ $\begin{array}{c} \left.{\text{sin}}^{3}{\theta^{c}}_{\text{max}}+\frac{3\mu_{\text{local}}}{2}-\text{ln}\left(\frac{1}{\text{cos}{\theta^{c}}_{\text{max}}}+\text{tan}{\theta^{c}}_{\text{max}}\right)\right) \end{array}$, and a first-order series expansion is performed while neglecting the constant term. This truncation may contribute to underestimation of the slope in the predicted linear relationship between ${\mu_{s}}^{\text{max}}$ and Λ.

## References

[1] A. R. Hinkle, W. G. Nöhring, R. Leute, T. Junge, L. Pastewka, The emergence of small-scale self-affine surface roughness from deformation. Sci. Adv. 6, eaax0847 (2020). 32110722 10.1126/sciadv.aax0847 PMC7021500

[2] R. W. Carpick, The contact sport of rough surfaces. Science 359, 38 (2018). 29302004 10.1126/science.aaq1814

[3] R. Buzio, C. Boragno, F. Biscarini, F. Buatier De Mongeot, U. Valbusa, The contact mechanics of fractal surfaces. Nat. Mater. 2, 233–236 (2003). 12690395 10.1038/nmat855

[4] C. Yan, H. Y. Chen, P. Y. Lai, P. Tong, Statistical laws of stick-slip friction at mesoscale. Nat. Commun. 14, 6221 (2023). 37798284 10.1038/s41467-023-41850-1 PMC10556047

[5] F. C. Hsia, S. Franklin, P. Audebert, A. M. Brouwer, D. Bonn, B. Weber, Rougher is more slippery: How adhesive friction decreases with increasing surface roughness due to the suppression of capillary adhesion. Phys. Rev. Res. 3, 043204 (2021).

[6] B. Weber, T. Susina, T. Junge, L. Pastewka, A. M. Brouwer, D. Bonn, Molecular probes reveal deviations from Amontons’ law in multi-asperity frictional contacts. Nat. Commun. 9, 888 (2018). 29497030 10.1038/s41467-018-02981-y PMC5832787

[7] A. Sanner, N. Kumar, A. Dhinojwala, T. D. Jacobs, L. Pastewka, Why soft contacts are stickier when breaking than when making them. Sci. Adv. 10, eadl1277 (2024). 38446897 10.1126/sciadv.adl1277 PMC10917342

[8] K. Farain, D. Bonn, Predicting frictional aging from bulk relaxation measurements. Nat. Commun. 14, 3606 (2023). 37330517 10.1038/s41467-023-39350-3 PMC10276840

[9] L. Afferrante, G. Violano, D. Dini, How does roughness kill adhesion? J. Mech. Phys. Solids 181, 105465 (2023).

[10] S. Dalvi, A. Gujrati, S. R. Khanal, T. D. B. Jacobs, Linking energy loss in soft adhesion to surface roughness. Proc. Natl. Acad. Sci. U.S.A. 116, 25484–25490 (2019). 31772024 10.1073/pnas.1913126116 PMC6925979

[11] J. Li, T. Kim, N. Lapusta, E. Biondi, Z. Zhan, The break of earthquake asperities imaged by distributed acoustic sensing. Nature 620, 800–806 (2023). 37532935 10.1038/s41586-023-06227-w

[12] A. Okamoto, E. Vinis, Oscillations in fluid pressure caused by silica precipitation in a fracture. Nat. Commun. 16, 1791 (2025). 39979335 10.1038/s41467-025-57199-6 PMC11842768

[13] S. Gvirtzman, D. S. Kammer, M. Adda-Bedia, J. Fineberg, How frictional ruptures and earthquakes nucleate and evolve. Nature 637, 369–374 (2025). 39780013 10.1038/s41586-024-08287-y

[14] D. Wang, H. Hu, S. Li, H. Tian, W. Fan, X. Li, X. Chen, A. C. Taylor, J. Shao, Sensing-triggered stiffness-tunable smart adhesives. Sci. Adv. 9, eadf4051 (2023). 36921055 10.1126/sciadv.adf4051 PMC10017039

[15] C. Linghu, Y. Liu, Y. Y. Tan, J. Heng, M. Sing, Y. Tang, A. Zhou, X. Wang, D. Li, H. Gao, K. J. Hsia, Overcoming the adhesion paradox and switchability conflict on rough surfaces with shape-memory polymers. Proc. Natl. Acad. Sci. U.S.A. 120, e2221049120 (2023). 36940332 10.1073/pnas.2221049120 PMC10068835

[16] Q. Meng, H. Song, Y. Zhou, X. Liu, X. Shi, Unifying linear proportionality between real contact area and load in rough surface contact. J. Mech. Phys. Solids 196, 105975 (2025).

[17] H. Terwisscha-Dekker, A. M. Brouwer, B. Weber, D. Bonn, Elastic contact between rough surfaces: Bridging the gap between theory and experiment. J. Mech. Phys. Solids 188, 105676 (2024).

[18] J. A. Greenwood, J. P. Williamson, Contact of nominally flat surfaces. Proc. R. Soc. Lond. A Math. Phys. Sci. 295, 300–319 (1966).

[19] C. P. Hsu, S. N. Ramakrishna, M. Zanini, N. D. Spencer, L. Isa, Roughness-dependent tribology effects on discontinuous shear thickening. Proc. Natl. Acad. Sci. U.S.A. 115, 5117–5122 (2018). 29717043 10.1073/pnas.1801066115 PMC5960318

[20] J. Wang, A. Tiwari, I. Sivebaek, B. Persson, Sphere and cylinder contact mechanics during slip. J. Mech. Phys. Solids 143, 104094 (2020). 10.1103/PhysRevE.102.043002 33212665

[21] E. Milanese, T. Brink, R. Aghababaei, J. F. Molinari, Emergence of self-affine surfaces during adhesive wear. Nat. Commun. 10, 1116 (2019). 30850605 10.1038/s41467-019-09127-8 PMC6408517

[22] B. Weber, T. Suhina, A. Brouwer, D. Bonn, Frictional weakening of slip interfaces. Sci. Adv. 5, eaav7603 (2019). 30972367 10.1126/sciadv.aav7603 PMC6450692

[23] D. Petrova, B. Weber, C. Allain, P. Audebert, C. H. Venner, A. Brouwer, D. Bonn, Fluorescence microscopy visualization of the roughness-induced transition between lubrication regimes. Sci. Adv. 5, eaaw4761 (2019). 31840054 10.1126/sciadv.aaw4761 PMC6897541

[24] S. Akarapu, T. Sharp, M. O. Robbins, Stiffness of contacts between rough surfaces. Phys. Rev. Lett. 106, 204301 (2011). 21668231 10.1103/PhysRevLett.106.204301

[25] Z. Gao, W. Fu, W. Wang, W. Kang, Y. Liu, The study of anisotropic rough surfaces contact considering lateral contact and interaction between asperities. Tribol. Int. 126, 270–282 (2018).

[26] A. Misra, Effect of asperity damage on shear behavior of single fracture. Eng. Fract. Mech. 69, 1997–2014 (2002).

[27] S. Huang, A. Misra, Micro–macro-shear-displacement behavior of contacting rough solids. Tribol. Lett. 51, 431–436 (2013).

[28] S. Huang, D. Wei, W. Han, H. Song, S. Song, Y. Gan, C. Zhai, M. Xu, In-situ measurements of contact evolution for fractal rough surfaces under normal compression. Int. J. Solids. Struct. 297, 112841 (2024).

[29] D. Wei, C. Zhai, H. Song, R. Hurley, S. Huang, Y. Gan, M. Xu, Frictional Contacts Between Rough Grains With Fractal Morphology. J. Geophys. Res. Solid Earth 129, e2023JB028361 (2024).

[30] O. Ben-David, S. M. Rubinstein, J. Fineberg, Slip-stick and the evolution of frictional strength. Nature 463, 76–79 (2010). 20054393 10.1038/nature08676

[31] F. Barras, R. Aghababaei, J. F. Molinari, Onset of sliding across scales: How the contact topography impacts frictional strength. Phys. Rev. Mater. 5, 023605 (2021).

[32] A. Aymard, E. Delplanque, D. Dalmas, J. Scheibert, Designing metainterfaces with specified friction laws. Science 383, 200–204 (2024). 38207035 10.1126/science.adk4234

[33] T. Sato, Z. B. Milne, M. Nomura, N. Sasaki, R. W. Carpick, H. Fujita, Ultrahigh strength and shear-assisted separation of sliding nanocontacts studied in situ. Nat. Commun. 13, 2551 (2022). 35538085 10.1038/s41467-022-30290-y PMC9091249

[34] K. Sirorattanakul, S. Larochelle, V. Rubino, N. Lapusta, A. J. Rosakis, Sliding and healing of frictional interfaces that appear stationary. Nature 639, 947–953 (2025). 40074898 10.1038/s41586-025-08673-0

[35] A. Papangelo, J. Scheibert, R. Sahli, G. Pallares, M. Ciavarella, Shear-induced contact area anisotropy explained by a fracture mechanics model. Phys. Rev. E. 99, 053005 (2019). 31212526 10.1103/PhysRevE.99.053005

[36] R. Sahli, G. Pallares, A. Papangelo, M. Ciavarella, C. Ducottet, N. Ponthus, J. Scheibert, Shear-induced anisotropy in rough elastomer contact. Phys. Rev. Lett. 122, 214301 (2019). 31283347 10.1103/PhysRevLett.122.214301

[37] J. Lengiewicz, M. Souza, M. A. Lahmar, C. Courbon, D. Dalmas, S. Stupkiewicz, J. Scheibert, Finite deformations govern the anisotropic shear-induced area reduction of soft elastic contacts. J. Mech. Phys. Solids 143, 104056 (2020).

[38] O. Ben-David, J. Fineberg, Static friction coefficient is not a material constant. Phys. Rev. Lett. 106, 254301 (2011). 21770644 10.1103/PhysRevLett.106.254301

[39] S. Shi, M. Wang, Y. Poles, J. Fineberg, How frictional slip evolves. Nat. Commun. 14, 8291 (2023). 38092832 10.1038/s41467-023-44086-1 PMC10719317

[40] S. Yao, H. Yang, Rupture phases reveal geometry-related rupture propagation in a natural earthquake. Sci. Adv. 11, eadq0154 (2025). 39841824 10.1126/sciadv.adq0154 PMC11753371

[41] G. Volpe, C. Collettini, J. Taddeucci, C. Marone, G. Pozzi, Frictional instabilities in clay illuminate the origin of slow earthquakes. Sci. Adv. 10, eadn0869 (2024). 38941467 10.1126/sciadv.adn0869 PMC11212734

[42] B. N. J. Persson, On the fractal dimension of rough surfaces. Tribol. Lett. 54, 99–106 (2014).

[43] B. Fu, R. M. Espinosa-Marzal, Velocity-weakening and-strengthening friction at single and multiasperity contacts with calcite single crystals. Proc. Natl. Acad. Sci. U.S.A. 119, e2112505119 (2022). 35613057 10.1073/pnas.2112505119 PMC9295777

[44] R. W. Liefferink, B. Weber, C. Coulais, D. Bonn, Geometric control of sliding friction. Extreme. Mech. Lett. 49, 101475 (2021).

[45] P. R. Nayak, Random process model of rough surfaces in plastic contact. Wear 26, 305–333 (1973).

[46] W. B. Dapp, A. Lücke, B. N. Persson, M. H. Müser, Self-affine elastic contacts: Percolation and leakage. Phys. Rev. Lett. 108, 244301 (2012). 23004275 10.1103/PhysRevLett.108.244301

[47] J. H. Dieterich, B. D. Kilgore, Imaging surface contacts: Power law contact distributions and contact stresses in quartz, calcite, glass and acrylic plastic. Tectonophysics 256, 219–239 (1996).

[48] J. M. Monti, L. Pastewka, M. O. Robbins, Fractal geometry of contacting patches in rough elastic contacts. J. Mech. Phys. Solids 160, (2022).

[49] D. Bi, J. Zhang, B. Chakraborty, R. P. Behringer, Jamming by shear. Nature 480, 355–358 (2011). 22170683 10.1038/nature10667

[50] O. Hod, E. Meyer, Q. Zheng, M. Urbakh, Structural superlubricity and ultralow friction across the length scales. Nature 563, 485–492 (2018). 30464268 10.1038/s41586-018-0704-z

[51] L. Pastewka, M. O. Robbins, Contact between rough surfaces and a criterion for macroscopic adhesion. Proc. Natl. Acad. Sci. U.S.A. 111, 3298–3303 (2014). 24550489 10.1073/pnas.1320846111 PMC3948287

[52] X. Liang, M. Wang, C. Jiang, S. Wang, C. Li, G. Wang, Experimental studies on the interfacial separation and stiffness of rough elastic-plastic solids. Int. J. Solid. Struct. 296, 112846 (2024).

[53] C. Marone, Laboratory-derived friction laws and their application to seismic faulting. Annu. Rev. Earth Planet. Sci. 26, 643–696 (1998).

[54] A. Ruina, Slip instability and state variable friction laws. J. Geophys. Res. Solid Earth. 88, 10359–10370 (1983).

[55] N. Beeler, T. Tullis, J. Weeks, The roles of time and displacement in the evolution effect in rock friction. Geophys. Res. Lett. 21, 1987–1990 (1994).

[56] T. Putelat, J. H. Dawes, J. R. Willis, On the microphysical foundations of rate-and-state friction. J. Mech. Phys. Solids 59, 1062–1075 (2011).

[57] A. Misra, Mechanistic model for contact between rough surfaces. J. Eng. Mech. 123, 475–484 (1997).

[58] D. A. Hanaor, Y. Gan, I. Einav, Contact mechanics of fractal surfaces by spline assisted discretisation. Int.J. Solids. Struct. 59, 121–131 (2015).

[59] K. Uchida, J. Honda, K. Y. Yoon, An algorithm for rough surface generation with inhomogeneous parameters. J. Algorithms. Comput. 5, 259–271 (2011).

[60] V. Bakolas, Numerical generation of arbitrarily oriented non-Gaussian three-dimensional rough surfaces. Wear 254, 546–554 (2003).

[61] J. C. Yoo, T. H. Han, Fast normalized cross-correlation. Circ. Syst. Signal Pr. 28, 819–843 (2009).

[62] C. Zhai, E. B. Herbold, R. C. Hurley, The influence of packing structure and interparticle forces on ultrasound transmission in granular media. Proc. Natl. Acad. Sci. U.S.A. 117, 16234–16242 (2020). 32601178 10.1073/pnas.2004356117 PMC7368278

[63] T. Y. Goh, S. N. Basah, H. Yazid, M. J. A. Safar, F. S. A. Saad, Performance analysis of image thresholding: Otsu technique. Measurement 114, 298–307 (2018).

[64] V. P. Singh, D. K. Frevert, *Watershed Models* (CRC Press, 2010).

[65] V. Klema, A. Laub, The singular value decomposition: Its computation and some applications. IEEE Trans Automat Contr 25, 164–176 (1980).

[66] Q. Sun, J. Zheng, Two-dimensional and three-dimensional inherent fabric in cross-anisotropic granular soils. Comput. Geotech. 116, 103197 (2019).

[67] C. S. Chang, A. Misra, Packing structure and mechanical properties of granulates. J. Eng. Mech. 116, 1077–1093 (1990).

[68] Z. Hu, W. Lu, M. Thouless, Slip and wear at a corner with Coulomb friction and an interfacial strength. Wear 338, 242–251 (2015).

[69] X. Shi, Y. Zou, H. Fang, Numerical investigation of the three-dimensional elastic–plastic sloped contact between two hemispheric asperities. J. Appl. Mech. 83, 101004 (2016).

[70] A. Misra, S. Huang, Micromechanical stress–displacement model for rough interfaces: Effect of asperity contact orientation on closure and shear behavior. Int. J. Solids. Struct. 49, 111–120 (2012).

