# Supplementary material for: Frictional strength regulated by roughness alignment
Source: Sci Adv. 2025 Sep 17;11(38):eady6779. doi: 10.1126/sciadv.ady6779 (PMC12442864; doi:10.1126/sciadv.ady6779)
Supplement: Supplementary file 1 — Supplementary Text S1 to S6 Figs. S1 to S16 Tables S1 to S20 [file sciadv.ady6779_sm.pdf]

Science Advances
AAAS

Supplementary Materials for

Frictional strength regulated by roughness alignment

Shaoqi Huang *et al.*

Corresponding author: Chongpu Zhai, zhaichongpu@xjtu.edu.cn; Minglong Xu, mlxu@xjtu.edu.cn

Sci. Adv. **11**, eady6779 (2025)
DOI: 10.1126/sciadv.ady6779

This PDF file includes:

Supplementary Text S1 to S6
Figs. S1 to S16
Tables S1 to S20

Supplementary Text

Section S1. Sample characterization

These contact pairs can be divided into three groups depending on the contact type and interfacial porosity under identical normal compression: (i) the flat-to-rough contact, i.e., S_1; (ii) randomly rough-to-rough contacts with relatively low roughness matching degrees, represented by S_2, S_3 and S_4; and (iii) rough-to-rough contacts with a high degree of roughness matching, including S_5, S_6, S_7 and S_8. For Gaussian surfaces, the surface geometry is controlled by two roughness parameters: the root-mean-square roughness R_{rms} and the autocorrelation length β_0, which primarily govern the vertical and horizontal roughness characteristics, respectively. In contrast, for fractal surfaces, the multiscale structure is generated by tuning the fractal dimension D_f and the roll-off wavelength $\lambda_{roll-off}$. As summarized in Supplementary Table 1, S_1 features a flat bottom surface with the R_{rms} value three orders of magnitude lower than that of the top rough counterpart. For S_2, the top and bottom surfaces exhibit the same β_0 but with a different R_{rms}. The same R_{rms}, but different autocorrelation length β_0 is designed for S_3. The top and bottom rough surfaces for S_4 are extracted from a well-mated interface with superimposed small random perturbations on both counterparts. Contact pairs of S_5 and S_6 are perfectly-mated contacts, with S_6 exhibiting greater roughness amplitude than S_5. Notably, we define the contact matching angle, χ, as the deviation from $\chi = 0°$ for the perfectly aligned interface. In this context, counterclockwise rotation is considered positive, while clockwise rotation is regarded as negative when viewed from above. Therefore, the extra sample, $S_6^{\chi=2°}$ indicates the modified contact state of S_6 with an applied counterclockwise rotation of the top surface by 2° about the z^g-axis.

Section S2. Generation of self-affine rough surfaces

The fractal rough surfaces are numerically generated using a standard Fourier filtering method (*61*), in which the surface height field is constructed to exhibit a power-law power spectral density (PSD) of the form,

$$C(q) = C_0 \left(\frac{q}{q_0}\right)^{-2(1+H_{urst})} \tag{S1}$$

where q is the wave number, q_0 is the minimum wave number, C_0 is the amplitude, and H_{urst} is the Hurst exponent, which is related to the fractal dimension $D_f = 3 - H_{urst}$. In this study, the generated surfaces are characterized by the following parameters: root-mean-square roughness R_{rms}, Hurst exponent H_{urst}, roll-off wavelength $\lambda_{roll-off}$, and cut-off wavelength $\lambda_{cut-off}$. The fractal dimension $D_f = 3 - H_{urst}$ is selected to be approximately 2.15 to reflect realistic surface conditions (*43*), while the cut-off wavelength $\lambda_{cut-off}$ is set to 10 μm, consistent with the resolution of the 3D printing process. The root-mean-square roughness is set to $R_{rms} = 120$ μm, matching the value used for the S_5 contact pairs in the main study.

Section S3. Transformation of microcontact local coordinates

To facilitate mechanical analysis, we introduced local coordinates ($o^L x^L y^L z^L$) at individual microcontact (Fig. 2b), and the local coordinate is obtained based on the singular value decomposition algorithm (*66*). The local coordinate system can be obtained from the global

coordinate system ($o^G x^G y^G z^G$) through a composite transformation sequence 'ZYX' and Euler rotation matrix $R_{ZYX}(\alpha, \beta, \gamma)$,

$$\begin{bmatrix} \boldsymbol{n}_x{}^G \\ \boldsymbol{n}_y{}^G \\ \boldsymbol{n}_z{}^G \end{bmatrix} = R_{ZYX}{}^T(\alpha, \beta, \gamma) \begin{bmatrix} \boldsymbol{n}_x{}^L \\ \boldsymbol{n}_y{}^L \\ \boldsymbol{n}_z{}^L \end{bmatrix}, \tag{S2}$$

where $R_{ZYX}(\alpha, \beta, \gamma)$ is equal to $R_Z(\alpha)R_Y(\beta)R_X(\gamma)$, $\alpha, \beta,$ and γ are the angles of rotation about the x^G, y^G, and z^G axes, respectively. $\begin{bmatrix} \boldsymbol{n}_x{}^G & \boldsymbol{n}_y{}^G & \boldsymbol{n}_z{}^G \end{bmatrix}^T$ are the unit vectors along the global axes and $\begin{bmatrix} \boldsymbol{n}_x{}^L & \boldsymbol{n}_y{}^L & \boldsymbol{n}_z{}^L \end{bmatrix}^T$ is the unit vectors along the local axes. Here, the Euler angle is obtained by the $R_{ZYX}(\alpha, \beta, \gamma)$,

$$\alpha = \arctan\left(\frac{-R_{23}{}^T}{R_{33}{}^T}\right), \tag{S3}$$

$$\beta = arccos\left(R_{13}{}^T\right), \tag{S4}$$

$$\gamma = \arctan\left(\frac{R_{12}{}^T}{R_{11}{}^T}\right). \tag{S5}$$

The probability density distribution of the Euler angles is presented in fig. S9. In addition, the contact orientation of each microcontact is defined by the zenith angle θ^c and azimuth angle φ^c in a global coordinate system, represented by the unit normal vector $\boldsymbol{n}^c$. The zenith angle θ^c has the following relationship,

$$\theta^c = \cos^{-1}\left([\boldsymbol{n}_z{}^L]^{\mathrm{T}} \cdot R_{ZYX}{}^T(\alpha, \beta, \gamma) \cdot \boldsymbol{n}_z{}^L\right). \tag{S6}$$

The azimuth angle φ_i can be determined using the projection operator, i.e.,

$$\varphi^c = \cos^{-1}\left([\boldsymbol{n}_z{}^L]^{\mathrm{T}} \cdot P \cdot R_{ZYX}{}^T(\alpha, \beta, \gamma) \cdot \boldsymbol{n}_z{}^L\right), \tag{S7}$$

where $P = \begin{bmatrix} 1 & 0 & 0 \\ 0 & 1 & 0 \\ 0 & 0 & 0 \end{bmatrix}$ is a projection transformation matrix.

Section S4. Asymptotic expansion of generalized extreme value (GEV) function

The Generalized Extreme Value (GEV) distribution is characterized by three crucial parameters: the position parameter μ, the scale parameter σ, and the shape parameter k. For the approximation case, the location parameter μ and scale parameter σ are approximately the mean and standard deviation of the variable x. In particular, the shape parameter k determines the tail shape of the GEV distribution (*4*). In the context of a specific distribution, both the peak region and the tail distribution warrant attention. Consequently, we examine the approximate form of the GEV distribution in these two cases independently. For the sake of discussion, we introduce the dimensionless variable $y = k(x - \mu)/\sigma$. The probability density function (PDF) $\phi(y)$ of Eq. 1 in the main text can be written as

$$\phi(y) = \frac{1}{\sigma} t(y)^{k+1} e^{-t(y)}, \tag{S8}$$

where $t(y) = (1 + y)^{-1/k}$. For an approximation of the tail distribution, i.e., $y \gg 1$ suggests a heavy tail, $t(y)$ can be simplified to $y^{-1/k}$ and brought to Eq. S8 to yield

$$\phi(y) \approx \frac{1}{\sigma} y^{-\frac{(k+1)}{k}} e^{-\mathrm{y}^{-\frac{1}{k}}}. \tag{S9}$$

Note that the exponent term $e^{-\mathrm{y}^{-1/\mathrm{k}}}$ rapidly approaches 1 as y increases and only its zero-term is considered, i.e.,

$$\phi(y) \approx \mathrm{y}^{-\frac{(k+1)}{k}}. \tag{S10}$$

The power-law form of Eq. S10 can be used to describe the distribution of microcontact areas. Specifically, $\phi(x) \propto x^{-\lambda}$, the decay exponent λ is only determined by the shape parameter $\lambda = (k+1)/k$, depending on the material, stress, and surface roughness. This scaling law is consistent with the results of rough surface contact in the simulations and experiments (*48, 49*). By incorporating the mean value of shape parameters for all contact pairs, we can derive the theoretically predicted value of decay exponent $\lambda = 2.22 \ \pm 0.17$. The calculated value is between 1.05 and 3, given in (*48*), slightly higher than the simulated value of 1.45. The deviation can be attributed to the influence of material plasticity (*49*).
Behavior near the peak indicates that the variable x approaches the position parameter μ or that the shape parameter k approaches zero, i.e., $|y| < 1$ corresponds to short or no tails. Thus, we are seeking a series expansion of $t(y)^{k+1} = (1 + y)^{-(k+1)/k}$ and take the first-order terms to yield,

$$t(y)^{k+1} \approx \left(1 - \frac{k+1}{k} y\right). \tag{S11}$$

Similarly, another term, $e^{-t(y)}$, can be expanded as a series,

$$e^{-t(y)} \approx \frac{1}{e}\left(1 + \frac{1}{k} y\right). \tag{S12}$$

Consequently, we obtain a lower-order approximation of $\phi(x)$ in the form of a parabola,

$$\phi(y) \approx \frac{1}{\sigma e}\left(1 - \frac{k+1}{k} y\right)\left(1 + \frac{1}{k} y\right). \tag{S13}$$

The location of the peak value can be calculated by the $x = -0.5k\sigma/(k+1) + \mu$, which is determined by position, scale, and shape parameters. For the aspect ratio of microcontact, the calculated value is $0.30 \ \pm 0.04$, which differs by only 11.7% from the parabolic fitting value of 0.34.

Section S5. Calculation of goodness-of-fit metrics

To evaluate the relative quality of candidate probability distributions fitted to experimental data (e.g., microcontact area, displacement, zenith angle), we employed the Akaike Information Criterion (AIC), a widely used metric in statistical model selection that balances model fit and complexity. For each model, the AIC is computed as

$$\mathrm{AIC}_i = 2k_i - 2\ln\left(\hat{L}_i\right), \tag{S14}$$

where k_i is the number of parameters in the model, and $\hat{L}_i$ is the maximum value of the likelihood function for the model given the observed data. After calculating AIC_i values for all candidate models, we compute the Akaike differences,

$$\Delta_i = \frac{\mathrm{AIC}_i - \min(\mathrm{AIC}_i)}{\max(\mathrm{AIC}_i)}. \tag{S15}$$

These differences are used to derive the relative Akaike weight w_i for each model,

$$w_i = \frac{\exp\left(-\frac{\Delta_i}{2}\right)}{\sum_j \exp\left(-\frac{\Delta_j}{2}\right)}, \tag{S16}$$

The Akaike weight w_i provides a normalized measure of the probability that the model is the best model among the candidates, given the data. In our analysis, this approach is applied to each dataset across different loading steps and contact configurations. The GEV distribution yields the highest Akaike weights in most cases (figs. S4–S7), indicating its superior performance in capturing the statistical behavior of microcontact quantities across rough interface evolution.

Section S6. Experiments for the rate- and state-dependent friction

Rate dependence

To assess the influence of sliding velocity on frictional response, we carried out a series of controlled-rate experiments spanning six orders of magnitude in velocity, ranging from 10^{-6} m/s to 10^{-2} m/s. This velocity range encompasses both quasi-static and low-speed dynamic regimes that are particularly relevant to stick-slip phenomena and frictional instabilities in rough surface contacts (*35*). Three representative contact configurations, all based on sample S_7 (fractal rough surfaces), are selected to represent low, medium, and high levels of roughness matching. The roughness matching angle is precisely controlled using a high-precision torsional motor, mounted on the top surface holder. To ensure repeatability and eliminate confounding effects such as wear or surface damage accumulation, each friction test is performed using a freshly fabricated contact pair, produced via high-resolution 3D printing. Each friction test is repeated five times under identical alignment and velocity conditions. All samples shared identical surface geometries to maintain experimental consistency across tests.

Sate dependence

The time-dependent evolution of interfacial properties was evaluated through a series of hold-slide experiments, designed to probe the role of the state variable in rough contact friction. For this purpose, we combined friction testing with in-situ 3D X-ray computed tomography (3DXRCT), enabling direct observation of microcontact structure evolution under controlled holding durations. These experiments aim to explore how contact "aging" processes affect the interfacial microstructure and, consequently, macroscopic frictional strength. Each test was performed under a constant nominal pressure of 2.93 MPa. The contact interface was held at rest for fixed durations of 0.5, 1, 2, 4, and 8 hours, allowing time-dependent interfacial processes. These holding periods allow time-dependent processes, such as asperity creep, contact growth, and interfacial structure reorganization. After each holding period, shear loading was applied at a constant displacement rate of 5 μm/s until the contact transitioned through the stick-slip regime into stable sliding, as illustrated in fig. S14a.

Supplementary Fig. S1

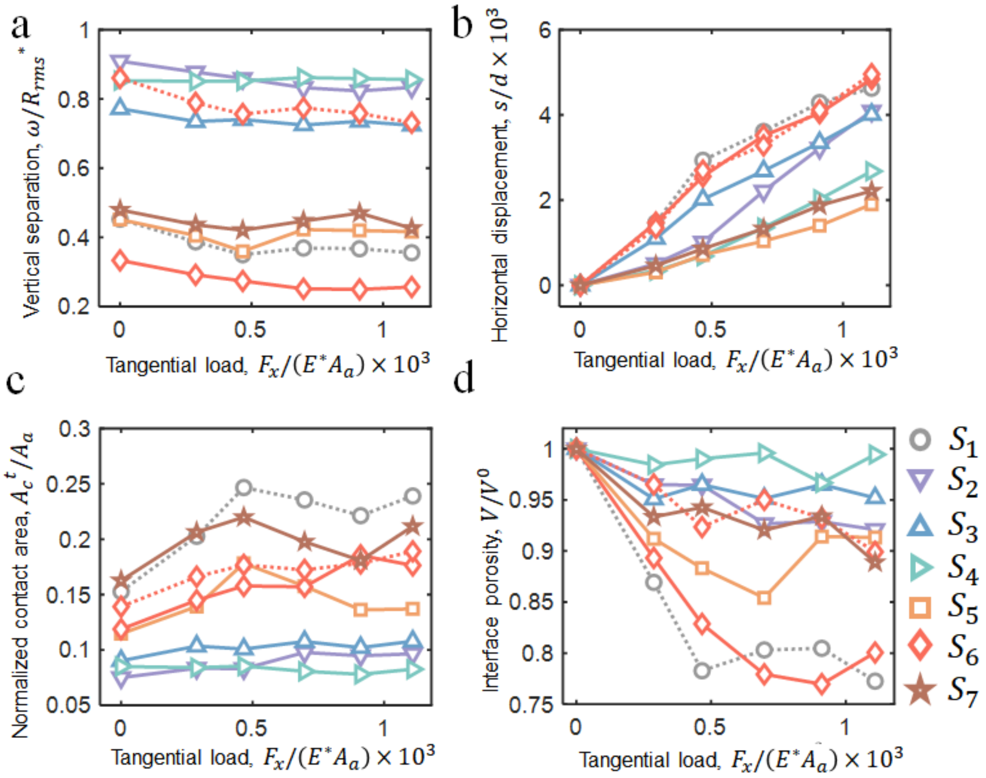

Fig. S1. Evolution of interface macro parameters under shear. (**a**) The normalized vertical separation distance, ω/d, defined as the relative distance between the mean height planes of the top and bottom rough surfaces. (**b**) The normalized horizontal displacement, s/d, determined by the displacement of the upper surface (the bottom surface is fixed during the shear tests). Here, d is the diameter of the top rough surface, and R_{rms}^* is the effective root mean square roughness, $R_{rms}^* = \sqrt{{R_{rms}^T}^2 + {R_{rms}^B}^2}$, where R_{rms}^T and R_{rms}^B are, respectively, the root mean square roughness of the top and bottom surfaces (*11*). (**c**) Evolutions of the normalized contact area, $A_c{}^t/A_a$. Here, $A_a = \pi d^2/4$ is the apparent contact area, and $E^* = 1/[(1-\nu_1{}^2)/E_1 + (1-\nu_2{}^2)/E_2]$ is the effective contact modulus, where E_1, ν_1 and E_2, ν_2 are Young's modulus and Poisson's ratio of the top and bottom surfaces. (**d**) The evolution of the interfacial porosity, V/V^0, calculated by the ratio of interfacial void volume with respect to that in the first load step. The gray dashed lines represent the behavior of the rough-flat contact, solid lines are for rough-rough contacts, and the red dashed line shows the contact pair $S_6{}^{\chi=2^\circ}$.

Supplementary Fig. S2.

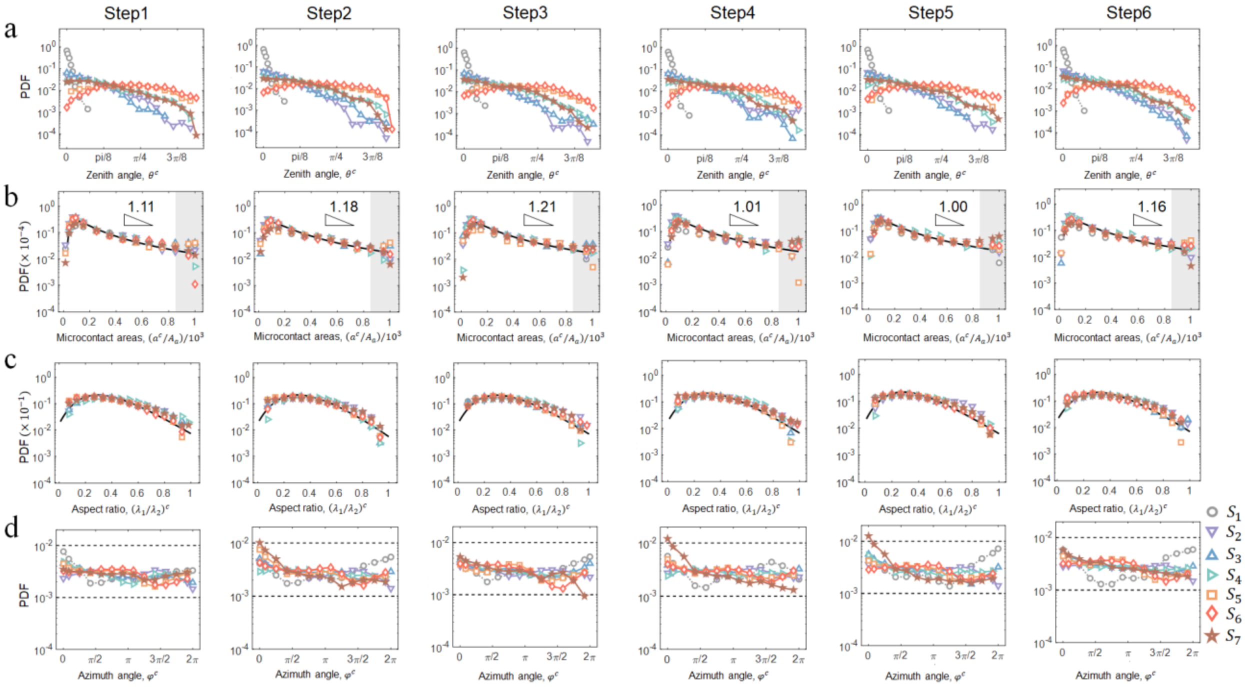

Fig. S2. The probability density distribution of micromechanical parameters. (a), (**b**), (**c**), and (**d**) correspond to the zenith angle, micro-contact area, aspect ratio, and azimuth angle, respectively. Different columns from left to right represent incremental load steps. The values of the decay exponent are calculated by the shape parameter, i.e., $\lambda = (k+1)/k$. The black lines in (**b**) and (**c**) fit GEV functions based on distributions for all samples. Obtained values of goodness of fitting are all higher than 0.95. The specific GEV fitting parameter values are presented in Supplementary Tables 2-19. The black dashed lines in (**d**) indicate that the probability distributions exhibit minimal fluctuations.

Supplementary Fig. S3.

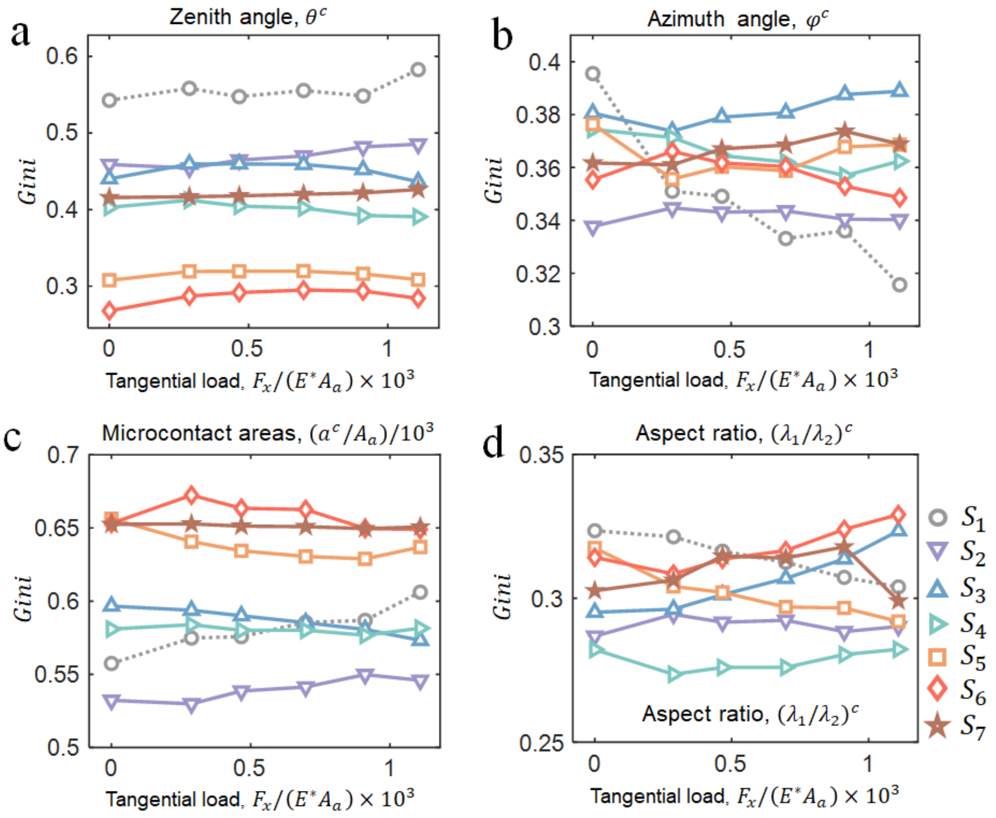

Fig. S3. The variation of the Gini coefficient of micromechanical parameters as the tangential load increases. (**a**), (**b**), (**c**), and (**d**) correspond to the zenith angle θ^c, azimuth angle φ^c, microcontact areas a^c/A_a, and aspect ratio $(\lambda_1/\lambda_2)^c$.

Supplementary Fig. S4.

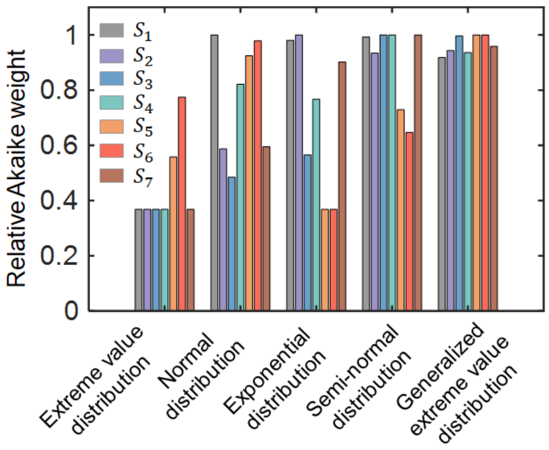

Fig. S4. Comparison of goodness-of-fit for different statistical functions applied to the zenith angle θ data.

Supplementary Fig. S5.

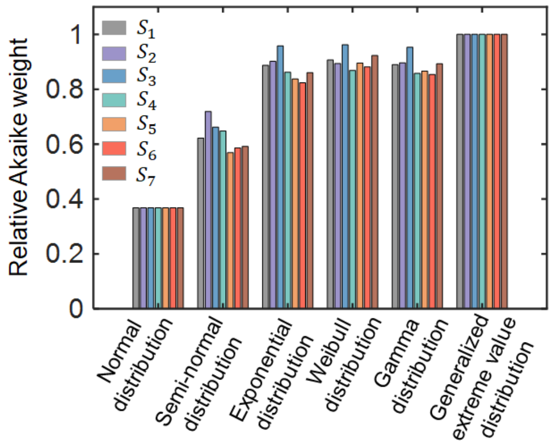

Fig. S5. Comparison of goodness-of-fit for different statistical functions applied to the microcontact areas a^c/A_a data.

Supplementary Fig. S6.

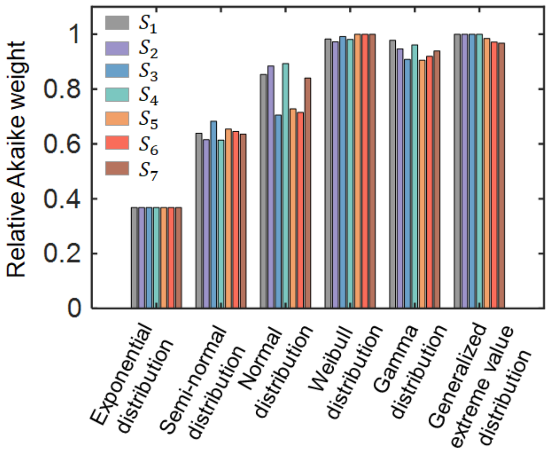

Fig. S6. Comparison of goodness-of-fit for different statistical functions applied to the aspect ratio λ_1/λ_2 data.

Supplementary Fig. S7.

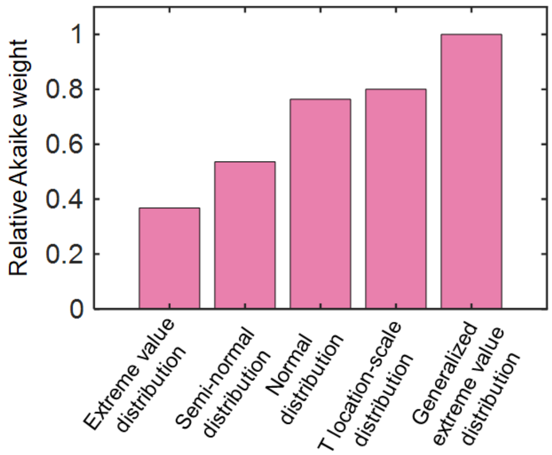

Fig. S7. Comparison of goodness-of-fit for different statistical functions applied to the relative microcontact deformation $|\Delta\boldsymbol{\delta}^c| = \left|(\Delta\delta_x, \Delta\delta_y, \Delta\delta_z)^g\right|$ data.

Supplementary Fig. S8.

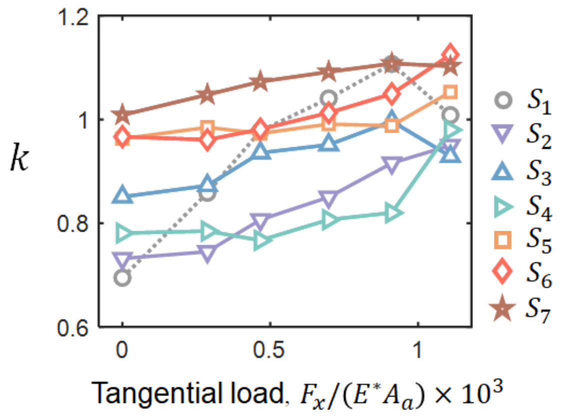

Fig. S8. The variation of the shape parameter k of the microcontact area with shear loads. More detailed data are presented in Supplementary Tables S2–S20.

Supplementary Fig. S9.

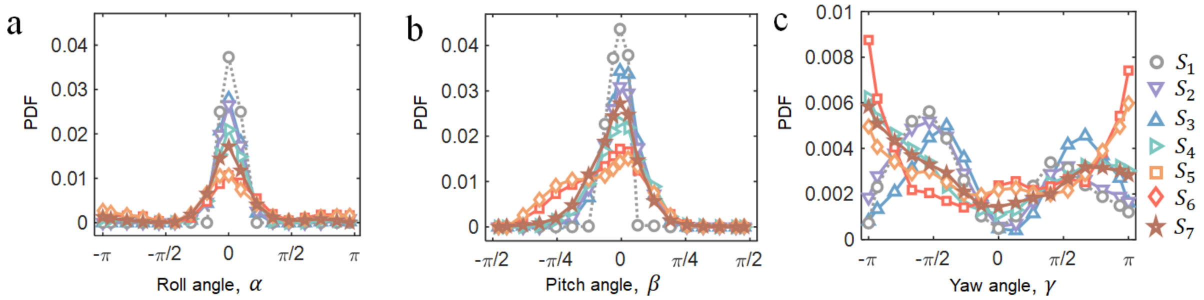

Fig. S9. The probability density distribution of the Euler angle. (**a**), (**b**), and (**c**) correspond to rotation angles α, β, and γ under normal compression, and the rotation order is "ZYX". The Euler rotation matrix $R_{ZYX}(\alpha, \beta, \gamma)$ can be expressed as the product of three rotation matrices, $R_Z(\alpha)R_Y(\beta)R_X(\gamma)$.

Supplementary Fig. S10.

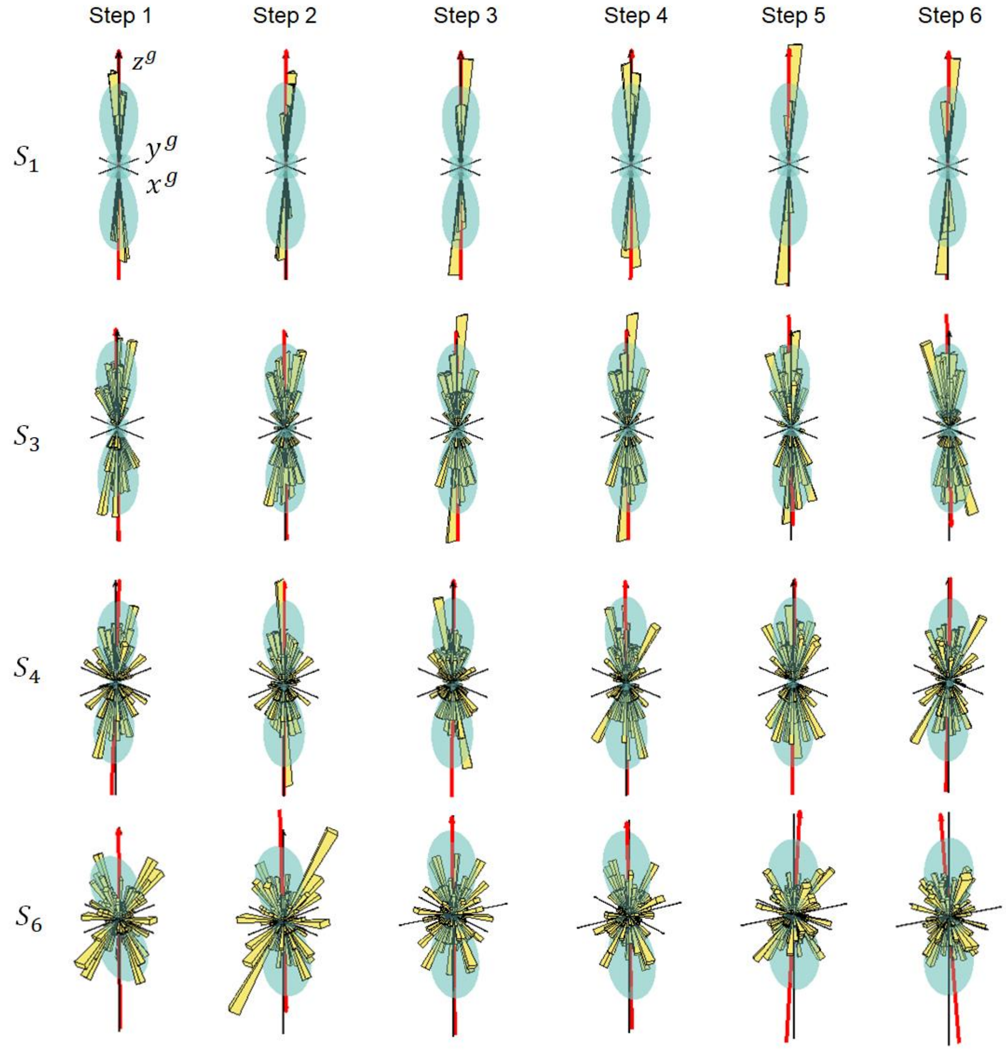

Fig. S10. 3D rose diagram of distributions for microcontact orientations. The yellow bars represent the discrete probability distribution of microcontact normals, and the cyan maps represent the continuous probability distribution $\mathrm{E}(\boldsymbol{n}^{\boldsymbol{c}})$. The solid red arrow pointing upwards is the principal direction of the contact fabric tensor and the solid black line represents the spatial 3D coordinates. Here, for contact pair S_1, the range of x and y is -0.01 to 0.01, and the range of z is -0.05 to 0.05; For contact pairs S_3, S_4 and S_6 , the range of x and y is -0.01 to 0.01, and the range of z is -0.015 to 0.015.

Supplementary Fig. S11.

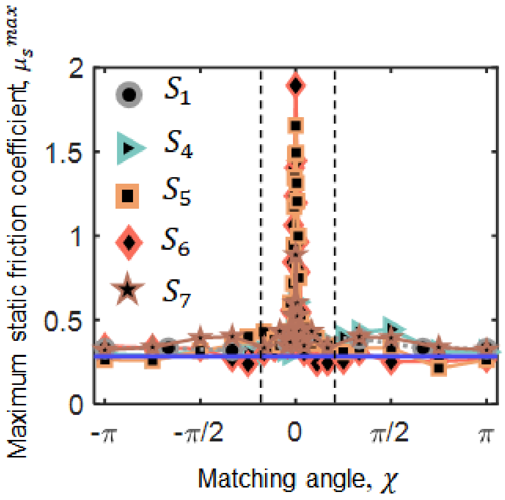

Fig. S11. The variation of the friction coefficient under different roughness matching angles. The solid blue line is the dynamic friction coefficient μ_d. Here, the region between the black dashed lines is enlarged in Fig. 4b of the main text.

Supplementary Fig. S12.

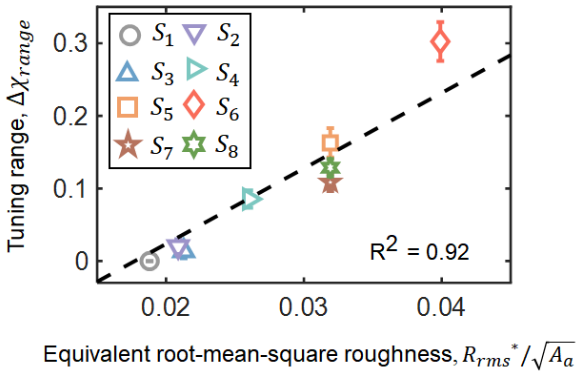

Fig. S12. The dependence of the tuning range $\Delta\chi_{range}$ on R^{*}_{rms}.

Supplementary Fig. S13.

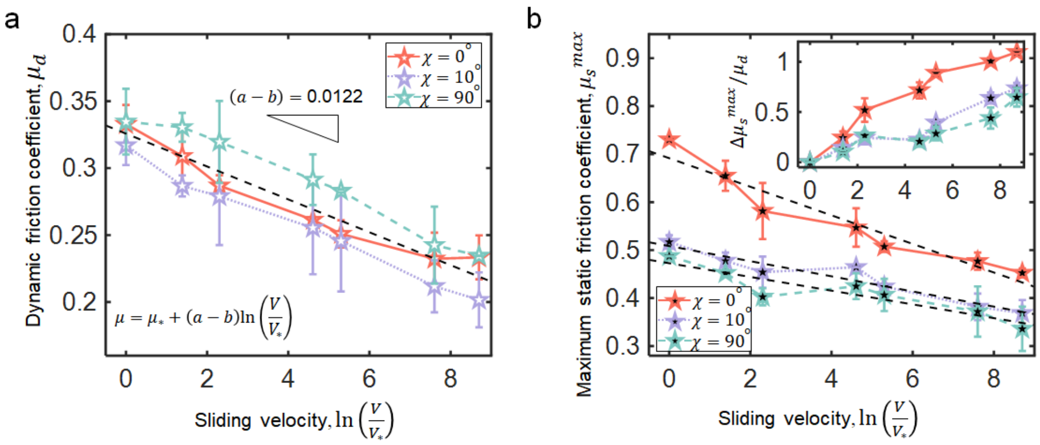

Fig. S13. The dependence of the friction coefficient on the sliding velocity (a) Dynamic friction coefficient μ_d of steady-state sliding with respect to sliding velocity. Here, μ_* is the friction coefficient at a reference velocity $V_* = 5\ \mu\text{m/s}$. (b) Maximum static friction coefficient ${\mu_s}^{max}$ with respect to sliding velocity. The insert shows the relationship between the ratio of the reduction in ${\mu_s}^{max}$ to μ_d and the sliding velocity. Here, the black dotted line represents the linear fitting, and the 0°, 10° and 90° angles respond to high, medium, and low roughness matching levels, respectively. Reported average values and their corresponding standard deviations are obtained over five measurements.

Supplementary Fig. S14.

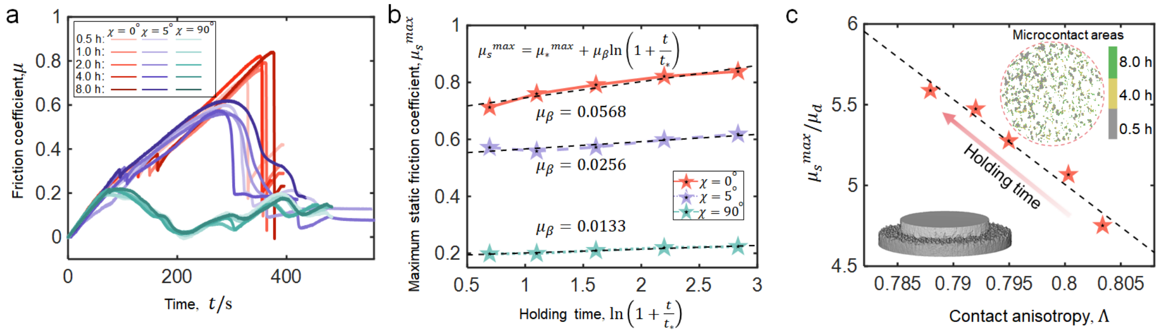

Fig. S14. The dependence of the friction coefficient on the holding time (a) The variation of the coefficient of friction of different matching angles over time. (b) The measured maximum static friction coefficient $\mu_s{}^{max}$ of contact pairs under constant normal compression load for various holding durations. (c) The relationship between the $\mu_s{}^{max}/\mu_d$ and the contact anisotropic index Λ. The insert in the lower left corner is the three-dimensional reconstructed topography. The insert in the upper right corner represents the evolution of the projection of the micro-contact area over time. Here, the black dotted line represents the linear fitting, and the 0°, 5° and 90° angles respond to high, medium, and low roughness matching levels, respectively.

Supplementary Fig. S15.

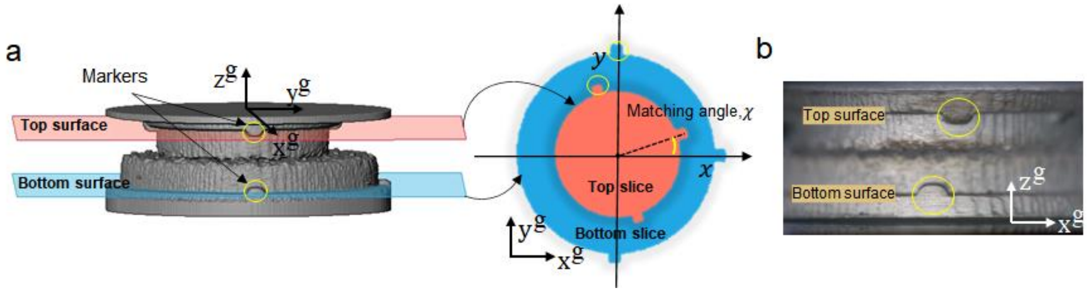

Fig. S15. Determination of the matching angle χ. (a) The 3D reconstruction of the top and bottom surfaces of sample S_4, and their top views are provided in the insert. The matching angle is calculated as the relative angle between the central lines of the top and bottom markers. (b) Photographs of the contact pair during friction testing.

Supplementary Fig. S16.

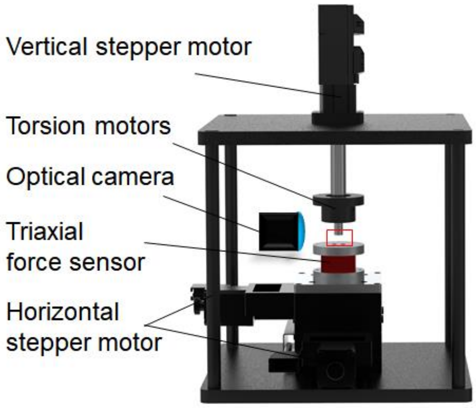

Fig. S16. Schematic of the self-developed friction testing system. The primary components consist of a stepper motor with a vertical motion resolution of 1 μm/s, a torsional motor with a resolution of 1 μrad, a high-precision CCD camera with a resolution of 0.1 μm, a triaxial force sensor with a sensitivity of 0.05 N, and a transverse stepper motor possessing a resolution of 1 μm/s.

Supplementary Table S1

Surface roughness characterizations of tested contact pairs, including root-mean-square roughness R_{rms}, slope of root-mean-square roughness R_{slope}, autocorrelation length β_0, roll-off wavelength λ_r, fractal dimension D_{box}, and interfacial void volume under normal loading.

No.		R_{rms} /μm	R_{slope}	β_0 /μm	λ_r /μm	D_{box}	Interfacial void volume V^0 /μm³
S_1	$S^T{}_1$	100	4.17 ± 0.003	50.0	340.0	2.65 ± 0.004	1.15×10^9
	$S^B{}_1$	0.24	$(0.07 \pm 0.001) \times 10^{-2}$	1000	1256.6	2.13 ± 0.009	
S_2	$S^T{}_2$	48.28	2.02 ± 0.006	50.0	184.8	2.65 ± 0.006	2.59×10^9
	$S^B{}_2$	100	4.18 ± 0.009	50.0	285.6	2.65 ± 0.006	
S_3	$S^T{}_3$	80	1.95 ± 0.008	100	314.2	2.56 ± 0.004	2.34×10^9
	$S^B{}_3$	80	3.43 ± 0.007	48.2	179.5	2.65 ± 0.005	
S_4	$S^T{}_4$	97.94	3.89 ± 0.007	53.7	287.8	2.65 ± 0.005	3.04×10^9
	$S^B{}_4$	97.92	3.88 ± 0.007	53.7	279.4	2.65 ± 0.005	
S_5	$S^T{}_5$	120	2.13 ± 0.010	150	392.7	2.48 ± 0.008	1.87×10^9
	$S^B{}_5$	120	2.13 ± 0.010	150	392.7	2.48 ± 0.008	
S_6	$S^T{}_6$	150	1.97 ± 0.007	200	523.4	2.42 ± 0.003	1.83×10^9
	$S^B{}_6$	150	1.97 ± 0.007	200	523.4	2.42 ± 0.003	
S_7	$S^T{}_7$	120	4.25 ± 0.006	48.8	20.0	2.15 ± 0.001	1.15×10^9
	$S^B{}_7$	120	4.25 ± 0.006	48.8	20.0	2.15 ± 0.001	
S_8	$S^T{}_8$	120	0.72 ± 0.005	557.2	2500	2.15 ± 0.001	0.50×10^9
	$S^B{}_8$	120	0.72 ± 0.005	557.2	2500	2.15 ± 0.001	

Supplementary Table S2.

Fitting parameters of the GEV distribution for the zenith angle θ^c under normal loading 83.06 N and tangential loading 0 N.

No.	k	σ	μ
S_1	0.241	0.598	0.475
S_2	0.403	6.222	7.411
S_3	0.374	5.603	6.447
S_4	0.492	9.685	11.165
S_5	0.465	23.808	27.252
S_6	0.366	24.250	29.699
S_7	0.180	11.521	13.736

Supplementary Table S3.

Fitting parameters of GEV distribution for the zenith angle θ^c under normal loading of 83.06 N and tangential loading of 6.53 N.

No.	k	σ	μ
S_1	0.360	0.566	0.432
S_2	0.490	6.546	5.970
S_3	0.564	7.278	7.134
S_4	0.457	11.332	11.775
S_5	0.559	22.169	18.816
S_6	0.336	28.545	31.398
S_7	0.089	11.821	13.671

Supplementary Table S4.

Fitting parameters of GEV distribution for the zenith angle θ^c under normal loading of 83.06 N and tangential loading of 10.60 N.

No.	k	σ	μ
S_1	0.013	0.676	0.538
S_2	0.418	6.814	6.680
S_3	0.453	6.730	6.418
S_4	0.458	11.590	11.750
S_5	0.443	21.631	22.536
S_6	0.322	27.174	31.696
S_7	0.150	11.082	12.438

Supplementary Table S5.

Fitting parameters of GEV distribution for the zenith angle θ^c under normal loading of 83.06 N and tangential loading of 15.85 N.

No.	k	σ	μ
S_1	3.855	0.012	0.003
S_2	0.493	7.802	7.351
S_3	0.457	6.965	6.613
S_4	0.462	11.603	12.150
S_5	0.563	23.416	23.427
S_6	0.362	26.212	30.018
S_7	0.061	11.980	13.773

Supplementary Table S6.

Fitting parameters of GEV distribution for the zenith angle θ^c under normal loading of 83.06 N and tangential loading of 20.70 N.

No.	k	σ	μ
S_1	5.116	0.288	0.056
S_2	0.517	6.935	6.345
S_3	0.424	7.039	7.145
S_4	0.457	12.152	12.357
S_5	0.449	25.240	28.398
S_6	0.329	26.327	30.102
S_7	0.178	11.558	12.960

Supplementary Table S7.

Fitting parameters of GEV distribution for the zenith angle θ^c under normal loading of 83.06 N and tangential loading of 25.20 N.

No.	k	σ	μ
S_1	5.154	0.081	0.016
S_2	0.587	7.192	6.431
S_3	0.495	7.541	7.865
S_4	0.414	11.975	12.705
S_5	0.464	24.426	27.787
S_6	0.306	25.579	30.998
S_7	0.098	12.250	13.382

Supplementary Table S8.

Fitting parameters of GEV distribution for the microcontact area a^c/A_a under normal loading 83.06 N and tangential loading 0 N.

No.	k	σ	μ
S_1	0.695	2.834×10^{-4}	2.821×10^{-4}
S_2	0.732	1.440×10^{-4}	1.529×10^{-4}
S_3	0.851	2.125×10^{-4}	2.024×10^{-4}
S_4	0.781	1.887×10^{-4}	1.920×10^{-4}
S_5	0.963	2.086×10^{-4}	1.875×10^{-4}
S_6	0.967	2.234×10^{-4}	2.002×10^{-4}
S_7	1.009	2.617×10^{-4}	2.467×10^{-4}

Supplementary Table S9.

Fitting parameters of GEV distribution for the microcontact area a^c/A_a under normal loading of 83.06 N and tangential loading of 6.53 N.

No.	k	σ	μ
S_1	0.840	3.547×10^{-4}	3.171×10^{-4}
S_2	0.681	1.626×10^{-4}	1.503×10^{-4}
S_3	0.983	2.163×10^{-4}	2.047×10^{-4}
S_4	0.971	1.643×10^{-4}	1.767×10^{-4}
S_5	1.195	2.393×10^{-4}	1.513×10^{-4}
S_6	1.165	2.232×10^{-4}	2.077×10^{-4}
S_7	1.022	3.260×10^{-4}	2.943×10^{-4}

Supplementary Table S10.

Fitting parameters of GEV distribution for the microcontact area a^c/A_a under normal loading of 83.06 N and tangential loading of 10.60 N.

No.	k	σ	μ
S_1	1.354	3.308×10^{-4}	2.485×10^{-4}
S_2	0.670	1.616×10^{-4}	1.559×10^{-4}
S_3	1.000	2.198×10^{-4}	1.993×10^{-4}
S_4	1.029	1.603×10^{-4}	1.651×10^{-4}
S_5	0.983	3.677×10^{-4}	2.814×10^{-4}
S_6	1.125	2.226×10^{-4}	2.131×10^{-4}
S_7	1.129	2.933×10^{-4}	2.622×10^{-4}

Supplementary Table S11.

Fitting parameters of GEV distribution for the microcontact area a^c/A_a under normal loading of 83.06 N and tangential loading of 15.85 N.

No.	k	σ	μ
S_1	5.240	5.596×10^{-4}	1.068×10^{-4}
S_2	1.110	1.321×10^{-4}	1.368×10^{-4}
S_3	1.006	2.401×10^{-4}	2.207×10^{-4}
S_4	0.755	1.876×10^{-4}	1.860×10^{-4}
S_5	0.940	3.421×10^{-4}	2.780×10^{-4}
S_6	1.105	2.166×10^{-4}	2.086×10^{-4}
S_7	1.123	2.837×10^{-4}	2.652×10^{-4}

Supplementary Table S12.

Fitting parameters of GEV distribution for the microcontact area a^c/A_a under normal loading of 83.06 N and tangential loading of 20.70 N.

No.	k	σ	μ
S_1	1.073	3.644×10^{-4}	2.996×10^{-4}
S_2	0.971	1.484×10^{-4}	1.540×10^{-4}
S_3	1.185	1.885×10^{-4}	1.993×10^{-4}
S_4	0.701	2.065×10^{-4}	1.840×10^{-4}
S_5	0.932	3.086×10^{-4}	2.535×10^{-4}
S_6	1.096	2.743×10^{-4}	2.577×10^{-4}
S_7	1.098	2.991×10^{-4}	2.634×10^{-4}

Supplementary Table S13.

Fitting parameters of GEV distribution for the microcontact area a^c/A_a under normal loading of 83.06 N and tangential loading of 25.20 N.

No.	k	σ	μ
S_1	1.201	3.322×10^{-4}	2.634×10^{-4}
S_2	0.950	1.435×10^{-4}	1.531×10^{-4}
S_3	0.929	2.492×10^{-4}	2.387×10^{-4}
S_4	0.980	1.643×10^{-4}	1.736×10^{-4}
S_5	1.053	2.688×10^{-4}	2.438×10^{-4}
S_6	1.125	2.553×10^{-4}	2.423×10^{-4}
S_7	1.103	2.972×10^{-4}	2.640×10^{-4}

Supplementary Table S14.

Fitting parameters of GEV distribution for the aspect ratio $(\lambda_1/\lambda_2)^c$ under normal loading 83.06 N and tangential loading 0 N.

No.	k	σ	μ
S_1	-0.020	0.174	0.271
S_2	-0.157	0.181	0.314
S_3	-0.084	0.182	0.306
S_4	-0.129	0.191	0.333
S_5	-0.011	0.163	0.261
S_6	-0.008	0.160	0.258
S_7	-0.009	0.166	0.287

Supplementary Table S15.

Fitting parameters of GEV distribution for the aspect ratio $(\lambda_1/\lambda_2)^c$ under normal loading of 83.06 N and tangential loading of 6.53 N.

No.	k	σ	μ
S_1	-0.007	0.1690	0.274
S_2	-0.238	0.207	0.311
S_3	-0.055	0.146	0.261
S_4	-0.171	0.165	0.325
S_5	-0.091	0.189	0.213
S_6	-0.029	0.157	0.274
S_7	-0.086	0.182	0.294

Supplementary Table S16.

Fitting parameters of GEV distribution for the aspect ratio $(\lambda_1/\lambda_2)^c$ under normal loading of 83.06 N and tangential loading of 10.60 N.

No.	k	σ	μ
S_1	-0.002	0.164	0.261
S_2	-0.125	0.206	0.301
S_3	-0.011	0.155	0.268
S_4	-0.267	0.188	0.333
S_5	-0.132	0.170	0.262
S_6	-0.009	0.161	0.275
S_7	-0.098	0.186	0.310

Supplementary Table S17.

Fitting parameters of GEV distribution for the aspect ratio $(\lambda_1/\lambda_2)^c$ under normal loading of 83.06 N and tangential loading of 15.85 N.

No.	k	σ	μ
S_1	0.158	0.182	0.145
S_2	-0.086	0.175	0.318
S_3	-0.042	0.158	0.264
S_4	-0.229	0.192	0.323
S_5	-0.106	0.166	0.264
S_6	-0.002	0.177	0.281
S_7	-0.021	0.176	0.273

Supplementary Table S18.

Fitting parameters of GEV distribution for the aspect ratio $(\lambda_1/\lambda_2)^c$ under normal loading of 83.06 N and tangential loading of 20.70 N.

No.	k	σ	μ
S_1	-0.064	0.156	0.262
S_2	-0.121	0.185	0.327
S_3	-0.025	0.150	0.251
S_4	-0.159	0.185	0.281
S_5	-0.102	0.172	0.267
S_6	-0.013	0.155	0.264
S_7	-0.053	0.178	0.273

Supplementary Table S19.

Fitting parameters of GEV distribution for the aspect ratio $(\lambda_1/\lambda_2)^c$ under normal loading of 83.06 N and tangential loading of 25.20 N.

No.	k	σ	μ
S_1	-0.062	0.175	0.279
S_2	-0.092	0.183	0.327
S_3	-0.034	0.156	0.245
S_4	-0.106	0.178	0.326
S_5	-0.054	0.151	0.274
S_6	-0.056	0.169	0.264
S_7	-0.116	0.186	0.313

Supplementary Table S20.

Fitting parameters of GEV distribution for the microcontact displacement $S_d(|\Delta\boldsymbol{\delta}_i|)$ between all adjacent loading steps. Here, to reasonably combine datasets from different loading steps and across all contacts, the data collected for contact at a given measurement step is normalized with the standard score, i.e., $S_d(X_i) = (X_i - \bar{X})/std(X)$. Here, X represents the dataset, $\bar{X}$ and $std(X)$ are the mean and standard deviation, respectively.

No.	k	σ	μ
S_1	0.129	0.632	-0.456
S_2	0.048	0.757	-0.478
S_3	0.068	0.741	-0.482
S_4	-0.057	0.836	-0.440
S_5	-0.047	0.824	-0.441
S_6	-0.094	0.861	-0.432
S_7	-0.062	0.837	-0.435
